# Terminal Differentiation of Adult Hippocampal Progenitor Cells Is a Step Functionally Dissociable from Proliferation and Is Controlled by Tis21, Id3 and NeuroD2

**DOI:** 10.3389/fncel.2017.00186

**Published:** 2017-07-10

**Authors:** Laura Micheli, Manuela Ceccarelli, Roberta Gioia, Giorgio D’Andrea, Stefano Farioli-Vecchioli, Marco Costanzi, Daniele Saraulli, Vincenzo Cestari, Felice Tirone

**Affiliations:** ^1^Institute of Cell Biology and Neurobiology, Consiglio Nazionale delle Ricerche (CNR), Fondazione Santa Lucia (IRCCS)Rome, Italy; ^2^Department of Human Sciences, Libera Università Maria SS. Assunta (LUMSA)Rome, Italy; ^3^Department of Psychology, Sapienza Università di RomaRome, Italy

**Keywords:** hippocampal neurogenesis, neural differentiation, neural progenitor cells, NEUROD2, Id3, Tis21, mouse models, neurogenic stimuli

## Abstract

Cell proliferation and differentiation are interdependent processes. Here, we have asked to what extent the two processes of neural progenitor cell amplification and differentiation are functionally separated. Thus, we analyzed whether it is possible to rescue a defect of terminal differentiation in progenitor cells of the dentate gyrus, where new neurons are generated throughout life, by inducing their proliferation and/or their differentiation with different stimuli appropriately timed. As a model we used the Tis21 knockout mouse, whose dentate gyrus neurons, as demonstrated by us and others, have an intrinsic defect of terminal differentiation. We first tested the effect of two proliferative as well as differentiative neurogenic stimuli, one pharmacological (fluoxetine), the other cognitive (the Morris water maze (MWM) training). Both effectively enhanced the number of new dentate gyrus neurons produced, and fluoxetine also reduced the S-phase length of Tis21 knockout dentate gyrus progenitor cells and increased the rate of differentiation of control cells, but neither factor enhanced the defective rate of differentiation. In contrast, the defect of terminal differentiation was fully rescued by *in vivo* infection of proliferating dentate gyrus progenitor cells with retroviruses either silencing Id3, an inhibitor of neural differentiation, or expressing NeuroD2, a proneural gene expressed in terminally differentiated dentate gyrus neurons. This is the first demonstration that NeuroD2 or the silencing of Id3 can activate the differentiation of dentate gyrus neurons, complementing a defect of differentiation. It also highlights how the rate of differentiation of dentate gyrus neurons is regulated genetically at several levels and that a neurogenic stimulus for amplification of neural stem/progenitor cells may not be sufficient in itself to modify this rate.

## Introduction

Cell proliferation and differentiation of neural stem and progenitor cells are correlated processes. It is known that the cell cycle machinery exerts a direct role in fate determination and in the first stages of differentiation (Hardwick et al., 2015) and, on the other hand, that several transcription factors coordinately control both differentiation and cell cycle exit by regulating the expression of several cell cycle components (Lacomme et al., 2012). Moreover, it has been proposed that variations of cell cycle length regulate differentiation progression (Calegari and Huttner, 2003; Calegari et al., 2005; Brandt et al., 2012). In fact, a positive or negative modulation of the expression of cyclin D1, which controls the G1 to S phase progression of the cell cycle, affects the abundance of progenitor cells (Lange et al., 2009). However, the amplification of neural stem cells triggered by cyclin D1 overexpression is not sufficient to enhance the generation of new neurons, unless the proliferative stimulus ceases after amplification, in order to allow the process of differentiation (Artegiani et al., 2011). This suggests that expansion and differentiation of neural stem cells are correlated processes, although the extent to which they are mutually interacting remains open to investigation.

We have recently observed that ablation of the antiproliferative transcriptional cofactor Tis21 (also known as PC3 or Btg2), although increasing the proliferation of progenitor cells of the dentate gyrus, paradoxically leads to accumulation of early differentiated neurons, which cannot terminally differentiate although they have already exited the cell cycle (Farioli-Vecchioli et al., 2009). We and others have demonstrated, by silencing Tis21, that Tis21 exerts on neural cells of the dentate gyrus and subventricular zone an intrinsic pro-differentiative action (Farioli-Vecchioli et al., 2009, 2014a; Attardo et al., 2010). This Tis21 knockout-dependent selective impairment of the terminal differentiation suggests that this step is functionally separable from the cell cycle.

The dentate gyrus of the hippocampus is a neurogenic niche where new neurons are generated throughout adulthood from putative neural stem cells with radial glial-like morphology, identified by the expression of GFAP in their processes (Seri et al., 2001), and expressing also nestin or Sox2 (defined as type-1 cells; Graham et al., 2003; Kempermann et al., 2004; Komitova and Eriksson, 2004). Stem cells mature through successive stages in proliferating progenitor cells, namely type-2ab and type-3 cells. Type-2a and type-2b are both nestin positive, but only the latter is positive for the immature neuronal marker doublecortin (DCX), whereas type-3 cells express mainly DCX (Filippov et al., 2003; Fukuda et al., 2003; Kronenberg et al., 2003). Finally, progenitor cells attain the early postmitotic stage of adult granule cell, characterized by the transient expression of Ca-binding protein Calretinin (stage 5), which is later replaced with Calbindin in mature granule cells (stage 6; Brandt et al., 2003; Steiner et al., 2004). The endogenous Tis21 is expressed in type-2/type-3 progenitor cells and in stage 6 (Calbindin-positive) terminally differentiated neurons, but is absent in stage 5 neurons (Attardo et al., 2010), with a pattern fully compatible with the stage 6-specific impairment of maturation observed after ablation of Tis21. In fact, in the absence of Tis21, stage 5 early postmitotic neurons cannot differentiate into stage 6 neurons (Farioli-Vecchioli et al., 2009).

In this report, our first aim has been to investigate the relationship between proliferation and differentiation in dentate gyrus adult neurons by analyzing whether the impairment of terminal differentiation, elicited by Tis21 knockout, can be reversed by a neurogenic stimulus inducing mainly proliferation of progenitor cells, such as the inhibitor of serotonin reuptake, fluoxetine. At the origin of our attempt was also the recent finding that the proliferating stem cells (type-1, GFAP^+^) or the committed progenitor cells (type-3, NeuroD1^+^) of the hippocampus, divide faster accelerating the S-phase (Brandt et al., 2012). The authors surmise the idea that once stem/progenitor cells are activated, then they rapidly divide and differentiate. This hypothesis is congruent with a previous “disposable stem cell” model, where a stem cell that has left the quiescent state then rapidly travels toward a differentiated state (Encinas et al., 2011); while it is in contrast with previous findings indicating that the pool of expanding neural stem and precursor cells in the cortex invests more time in S-phase for quality control of DNA replication (Arai et al., 2011).

Chronic treatment with fluoxetine efficiently stimulates neurogenesis by selective targeting of the serotoninergic pathway (Malberg et al., 2000; Santarelli et al., 2003; Encinas et al., 2006). Fluoxetine has a neurogenic action less dependent from non-specific systemic effects involving general metabolism, vasculogenesis etc., produced by other proliferative stimuli such as physical exercise (van Praag et al., 1999; Kempermann, 2008; Falone et al., 2012). We chose fluoxetine also because it is endowed with the abilities to accelerate the maturation of immature DCX^+^ cells (Wang et al., 2008) as well as to promote the survival of progenitor cells of the dentate gyrus (Encinas et al., 2006). Specific protocols were adopted in order to highligth not only proliferation, but also differentiation and survival.

We also sought to rescue the terminal differentiation defect by physiologically inducing the maturation of dentate gyrus neurons through a cognitive stimulus, by a specific paradigm of spatial learning acting on 1-week-old neurons, i.e., at the end of the proliferative phase, when the neurogenic effect of the task is more pronounced (Gould et al., 1999; Epp et al., 2013).

Finally, we attempted with a focused approach to genetically complement the defective terminal differentiation, i.e., by regulating the expression of two genes, one of which, the key inhibitor of proneural basic helix-loop-helix (bHLH) genes, Id3 (Lyden et al., 1999; Yokota, 2001), is strictly involved in the Tis21-dependent mechanisms, whereas the other, the proneural gene NeuroD2 (Schwab et al., 2000; Olson et al., 2001), is coexpressed with Tis21 in differentiating dentate gyrus cells.

In fact, we silenced *in vivo* Id3, which is negatively regulated by Tis21 in dentate gyrus cells (Farioli-Vecchioli et al., 2009), or we overexpressed NeuroD2.

Notably, stage 5 neurons express mainly NeuroD1, which has been implicated in the process of hippocampal determination and terminal differentiation (Liu et al., 2000; Schwab et al., 2000; Gao et al., 2009), while stage 6 mature neurons co-express with Tis21 and NeuN also NeuroD2 (Roybon et al., 2009; Attardo et al., 2010). This latter has been shown to induce the neural phenotype and to be involved in dentate gyrus development but not, so far, in the process of its terminal differentiation (Olson et al., 2001; Sugimoto et al., 2009; Ravanpay et al., 2010).

We show in this report that the defect of terminal differentiation of Tis21-null dentate gyrus neurons can be rescued genetically, either by NeuroD2 overexpression or Id3 silencing *in vivo*, but not by neurogenic stimuli acting mainly, though not exclusively, on proliferation, even accelerating the cell cycle. This suggests that the proliferative state may influence the amplification of progenitor cells but not their terminal differentiation rate.

## Materials and Methods

### Mouse Lines and Genotyping

The Tis21 knockout mice were of the C57BL/6 (B6) strain and had a replacement of the entire exon II of the Tis21 gene. Mutant mice had been generated previously, as described (Park et al., 2004). Genotyping of mice was routinely performed by polymerase chain reaction (PCR), using genomic DNA from tail tips, as described (Farioli-Vecchioli et al., 2009). Mice were maintained under standard specific-pathogen-free conditions; all animal procedures were carried out on male mice and completed in accordance with the Istituto Superiore di Sanita’ (Italian Ministry of Health; authorizations DM 442-2016-PR and DM 04/2013) and current European (directive 2010/63/EU) Ethical Committee guidelines.

### BrdU and Fluoxetine Treatment of Mice and Sample Preparation for Immunohistochemistry

Differentiated (28-day-old) neurons in the dentate gyrus were detected in Tis21 wild-type and knockout mice after treatment with five daily i.p. injections of bromodeoxyuridine (BrdU; 95 mg/kg b.wt., 10 μl/g b.wt.; Sigma–Aldrich, S.Louis, MO, USA) from P60 to P64, followed by perfusion at P88. In Morris water maze (MWM) experiments BrdU was administered at P60 (two daily injections, 95 mg/kg i.p) and analysis was performed 21 days later. Fluoxetine (10 mg/Kg b.wt., 10 μl/g b.wt.; Tocris Bioscience, Bristol, United Kingdom) or vehicle in control groups (sterilized water) was administered daily by i.p. injection to 2-month-old mice for 18 days (in proliferation and differentiation experiments) or, in experiments aimed to measure the Ts length, to 39-day-old mice for 21 days. Treatments are summarized in Supplementary Table S1. Brains were collected after transcardiac perfusion with 4% paraformaldehyde (PFA) in PBS—DEPC and kept overnight in PFA. Brains of 14-day-old mice were directly fixed by immersion in PFA. Afterwards, brains were equilibrated in sucrose 30% and cryopreserved at −80°C.

### Immunohistochemistry

Immunohistochemistry was performed on serial free-floating coronal sections cut at 40 μm thickness in a cryostat at −25°C from brains embedded in Tissue-Tek OCT (Sakura, Torrence, CA, USA). The sections were then processed immunohistochemically for multiple labeling with BrdU and other cellular markers using fluorescent methods.

Proliferating subpopulations of progenitor cells were identified using a goat polyclonal antibody against DCX (Santa Cruz Biotechnology, Santa Cruz, CA, USA; SC-8066; 1:300) and a mouse monoclonal against nestin (Millipore, Temecula, CA, USA; MAB353; 1:100).

BrdU incorporation was visualized by denaturing DNA through pretreatment of sections with 2N HCl 45 min at 37°C, followed by 0.1 M sodium borate buffer pH 8.5 for 10 min. The sections were then incubated overnight at 4°C with a rat monoclonal antibody against BrdU (AbD Serotech, Raleigh, NC, USA; MCA2060; 1:400), together with other primary antibodies, as indicated: rabbit polyclonal antibody against Calretinin (Swant, Bellinzona, Switzerland; 7699/4, 1:200), mouse monoclonal antibody raised against NeuN (Millipore, Temecula, CA, USA; MAB377; 1:300).

Proliferating, immature and terminally differentiated neurons infected with retroviruses were visualized by means of chicken polyclonal antibody against GFP (green fluorescent protein; Aves Labs, Tigard, OR, USA; GFP-1010; 1:100) together with either a rabbit monoclonal antibody against Ki67 (Thermo Scientific, Fremont, CA, USA; SP6; 1:150), or a rabbit polyclonal antibody against Calretinin (Swant, Bellinzona, Switzerland; 7699/4, 1:200) and a mouse monoclonal antibody raised against NeuN (Millipore, Temecula, CA, USA; MAB377; 1:300). The NeuroD1 antibody used in cell cycle studies was a goat polyclonal (R&D Systems, Minneapolis, MN, USA; AF2746; 1:200).

Secondary antibodies used to visualize the antigen were all, with the exception of the secondary anti-GFP, from Jackson ImmunoResearch (West Grove, PA, USA) as follows: a donkey anti-rat antiserum conjugated to tetramethylrhodamine isothiocyanate (TRITC) (BrdU), a donkey anti-rabbit antiserum conjugated to Cy2 (calretinin), a donkey anti-mouse antiserum conjugated to Alexa-647 (NeuN), a donkey anti-rabbit antiserum Cy3-conjugated (Ki67 and calretinin), a donkey anti-mouse Cy2-conjugated (nestin) and a donkey anti-goat Alexa 647-conjugated (DCX or NeuroD1). The secondary antibody to visualize GFP was an anti-chicken antiserum fluorescein-conjugated (Aves Labs). Nuclei were counterstained by Hoechst 33258 (Sigma–Aldrich; 1 μg/ml in PBS). Supplementary Table S1 summarizes the cell staining used in the different experiments.

Images of the immunostained sections were obtained by laser scanning confocal microscopy using a TCS SP5 microscope (Leica Microsystem) equipped with Laser Diode 405, Ar 488, HeNe 543, HeNe 633. Analyses were performed in sequential scanning mode to rule out cross-bleeding between channels and using combinations of three different lasers simultaneously.

### Thymidine Analog Detection

For BrdU detection, see “Immunohistochemistry” Section above. Concerning IdU and CldU immunohistochemistry, sections were washed five times in TBS (Tris-buffered saline), pretreated with 2N HCl for 10 min, then washed five times in TBS. Antibodies were incubated in TBS containing 5% normal donkey serum and 0.25% Triton X-100 (Vega and Peterson, 2005).

Halogenated thymidine analogs were detected as follows: for IdU using the mouse monoclonal anti-BrdU (BD Biosciences, San Jose, CA, USA; BD 44; 1:500); for CldU using the rat monoclonal anti-BrdU (AbD Serotec, Raleigh, NC, USA; MCA2060; 1:250). Antibody specificity was analyzed in mice that were injected with either IdU or CldU alone. Secondary antibodies used to visualize the antigens were a donkey anti-rat antiserum conjugated to TRITC (CldU) and a donkey anti-mouse antiserum conjugated to Cy2 (IdU), all from Jackson ImmunoResearch (West Grove, PA, USA).

### Cell Cycle Analysis

S-phase length, measured in hours (Ts), was obtained employing the equation developed by others (Vega and Peterson, 2005; Brandt et al., 2012). In order to measure Ts in 2-month-old and 2-week-old mice, we administrated IdU intraperitoneally (57.5 mg/kg in 0.02 N NaOH, 0.9% NaCl saline solution; MP Biomedicals, Solon, OH, USA), and 3 h later CldU (42.5 mg/kg b.wt. in 0.9% NaCl saline solution; Sigma–Aldrich). The injected volumes were adjusted to the body weight. Forty-five minutes after CldU, injected animals were killed for immunohistochemistry. The Ts of the NeuroD1^+^ progenitors was obtained through triple immunofluorescence (NeuroD1^+^IdU^+^CldU^+^).

### Quantification of Cell Numbers

Stereological analysis of the number of cells was performed on one-in-eight series of 40-μm free-floating coronal sections (320 μm apart), which were analyzed by confocal microscopy to count cells expressing the indicated marker throughout the whole rostrocaudal extent of the dentate gyrus. To obtain the total estimated number of cells within the dentate gyrus, positive for each of the indicated markers, the average number of positive cells per section was multiplied by the total number of 40-μm sections comprising the entire dentate gyrus (about 50–60 sections), as described (Gould et al., 1999; Jessberger et al., 2005; Kee et al., 2007; Farioli-Vecchioli et al., 2008, 2012). Thus, about seven sections per mouse and at least three animals per group were analyzed. Confocal single plane images and Z-stacks with orthogonal projections of the immunostained sections were obtained using a TCS SP5 confocal laser scanning microscope (Leica Microsystems).

In Figures 3D, 4D (fluoxetine and MWM experiments, respectively) cell numbers were calculated as ratios of stage 5 or stage 6 neurons to the total number of BrdU^+^ cells. The virus-infected cells (Figures 5–7) were calculated as percentage ratios between GFP^+^Ki67^+^ or GFP^+^Calretinin^±^NeuN^+^ cells and the total number of infected cells (GFP^+^) in each section, and then averaged. In each virus-injected mouse were analyzed at least 15 sections throughout the whole extent of the dentate gyrus; the minimum number of GFP^+^ cells identified per mouse was 12. Cell number analyses were performed manually by trained experimenters using the I.A.S. software to record positive cells (Delta Sistemi, Rome, Italy).

**Figure 1:**
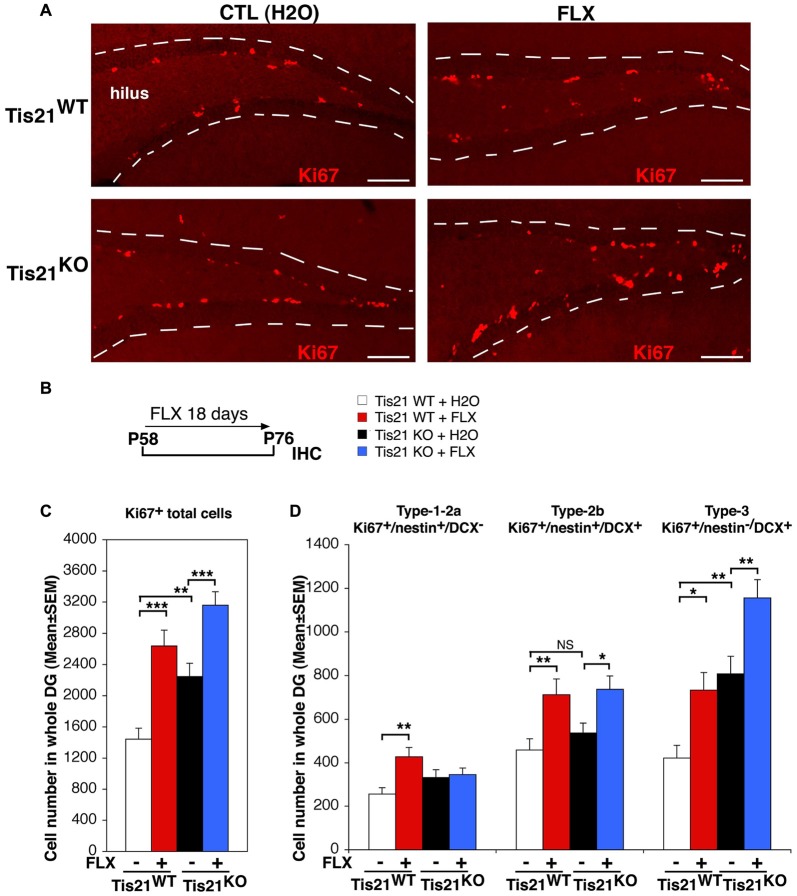
Treatment with fluoxetine significantly increases the number of dentate gyrus progenitor cells in adult (2-month-old) wild-type and Tis21 knockout mice. **(A)** Representative confocal images (20× magnification) showing proliferating dentate gyrus progenitor cells, labeled by Ki67 (red), in mice treated as described in **(B)**. Their number increases in Tis21^KO^ mice, as compared to Tis21^WT^ mice, and is increased also by fluoxetine in both the Tis21^KO^ and Tis21^WT^ genotypes, relative to the corresponding water-treated controls. The dotted lines define the outer boundary of the granule cell layer. Scale bar, 100 μm. **(B)** Scheme of the protocol followed: 2-month-old mice were treated daily with fluoxetine for 18 days and then analyzed. **(C)** Quantification of total proliferating adult progenitor cells in wild-type and Tis21 knockout dentate gyrus, measured as Ki67-positive cells; both were significantly increased by fluoxetine. Moreover, the ablation of Tis21 induced in itself an increase of total proliferating cells, relative to wild-type. Simple effects analysis: **p < 0.01, or ***p < 0.001; PLSD ANOVA test. **(D)** Quantification of individual proliferating stem and progenitor cell subpopulations in wild-type and Tis21^KO^ dentate gyrus, measured as Ki67-positive cells. The number of type-2b and type-3 progenitor cells is significantly induced by fluoxetine in both Tis21^WT^ and Tis21^KO^ mice. Simple effects analysis: *p < 0.05, **p < 0.01, or NS *p* > 0.05, PLSD ANOVA test. **(C,D)** Cell numbers in the dentate gyrus are mean ± SEM of the analysis of 4–6 animals per group (see Supplementary Table S1).

**Figure 2:**
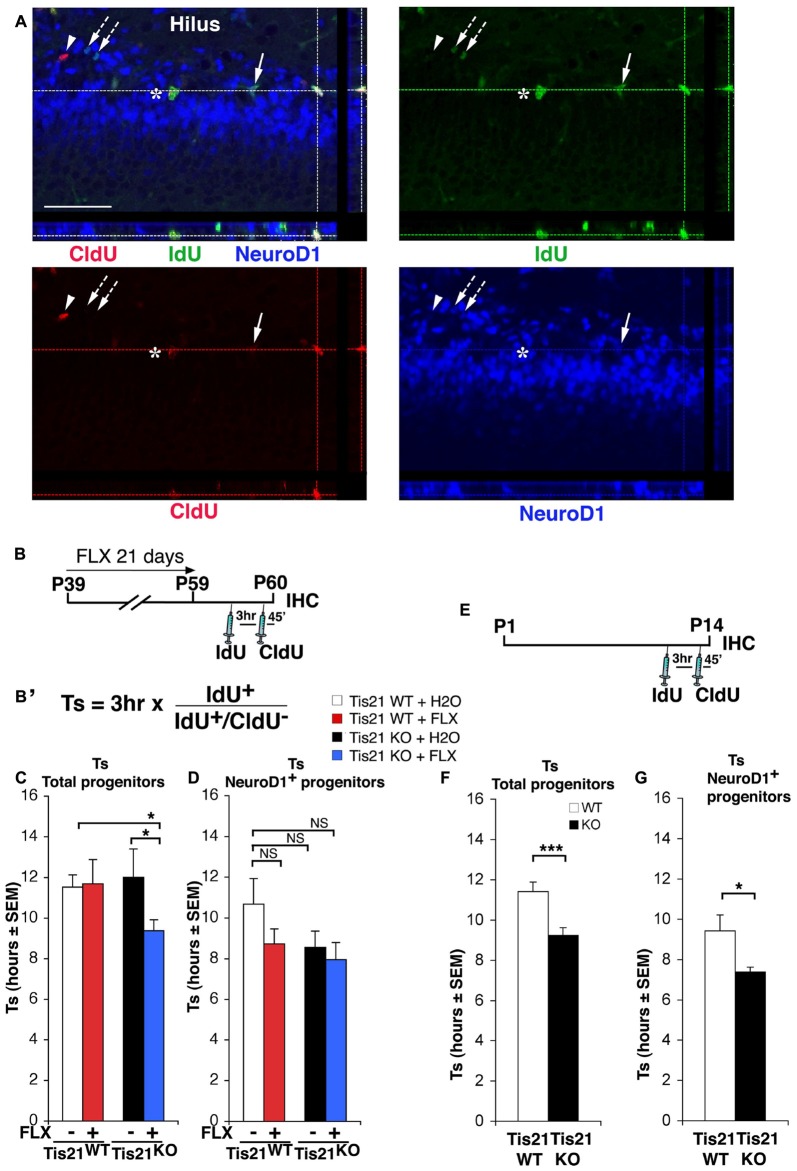
Treatment with fluoxetine significantly reduces the length of the S phase in Tis21 knockout 2-month-old dentate gyrus progenitor cells. **(A)** Representative confocal images of cycling cells (40× magnification), with 3D reconstruction from Z-stack and orthogonal projections of triple- and double-positive cells. Cycling cells are shown: (i) cells in S-phase during the 3 h interval after the IdU pulse and before CldU (IdU^+^/CldU^−^ cells, green, arrow; or NeuroD1^+^/IdU^+^/CldU^−^, blue-green, arrows with dotted line); (ii) cells in S-phase during the CldU pulse (IdU^−^/CldU^+^cells, red, arrowhead); (iii) or cells that were in S-phase during both pulses and continued throughout the cell cycle (IdU^+^/CldU^+^, yellow-green, asterisk; or NeuroD1^+^/IdU^+^/CldU^+^, white, at lines intersection). Scale bar, 50 μm. **(B)** Treatment timeline: 2-month-old wild-type and Tis21 knockout mice, after fluoxetine treatment for 21 days, were injected with IdU and CldU 3 h and 45 min, respectively, before analysis. **(B′)** Equation to calculate the S-phase length. The ratio of cells that have left the S phase during the interval of 3 h between injections (IdU^+^/CldU^−^ cells) to the total number of IdU^+^ cells is equal to 3 h/Ts. **(C)** Graphs showing the measurement of the length of the S phase (Ts) for the total population of progenitor cells and **(D)** for the NeuroD1^+^ progenitor cells population in the different experimental conditions. **(C)** A shortening of Ts is evident only in the total population of progenitor cells, in the Tis21^KO^ + FLX mice with respect to the Tis21^WT^ and Tis21^KO^ mice. Analysis of simple effects: *p < 0.05, PLSD ANOVA test. **(D)** NeuroD1^+^ progenitor cell population, analysis of simple effects: NS *p* > 0.05, PLSD ANOVA test. Data are mean ± SEM of the analysis of five **(C)** or 3–4 **(D)** animals per group (see Supplementary Table S1). **(E)** Treatment timeline and **(F)** analysis of S phase length (Ts) for the total proliferating stem/progenitor cells or **(G)** for the NeuroD1^+^ progenitor cells in 2-week-old wild-type and Tis21 knockout mice. *p < 0.05, or ***p < 0.001 Student’s *t* test. Data are mean ± SEM of the analysis of three animals per group.

**Figure 3:**
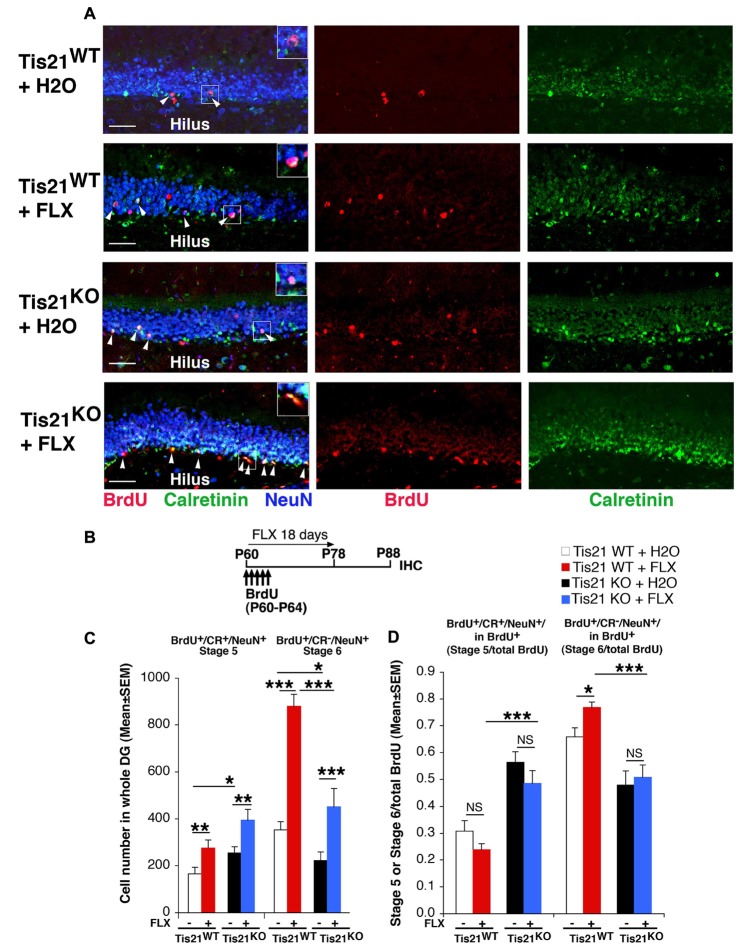
Treatment with fluoxetine highly increases the generation of early and late differentiated neurons in the adult dentate gyrus, but does not rescue the impairment of terminal differentiation in Tis21 knockout neurons. **(A)** Representative images (40× magnification) of the dentate gyrus of wild-type and mutant mice treated as in **(B)**, showing early postmitotic immature neurons (stage 5, identified as bromodeoxyuridine^+^ (BrdU^+^)/Calretinin^+^/NeuN^+^ cells; white arrowheads), and terminally differentiated neurons (stage 6 neurons, identified as BrdU^+^/Calretinin^−^/NeuN^+^ cells). Fluoxetine increases the absolute numbers of both stage 5 and stage 6 neurons in wild-type as well as Tis21 knockout dentate gyrus. Scale bar, 50 μm. The white box area is shown with 1.8× digital magnification. **(B)** Scheme of the protocol followed to detect new 28-day-old differentiated neurons: 2-month-old mice received five daily injection of BrdU at the beginning of the fluoxetine treatment, which was discontinued at day 18 for the following 10 days before analysis. **(C)** Quantification of the number of new 28-day-old cells indicates an increase in Tis21-null dentate gyrus of stage 5 immature neurons (BrdU^+^/Calretinin^+^/NeuN^+^) and a decrease of terminally differentiated stage 6 neurons (BrdU^+^/Calretinin^−^/NeuN^+^), relative to wild-type. Fluoxetine highly increases both stage 5 and stage 6 neurons in wild-type as well as Tis21 knockout dentate gyrus. Simple effects analysis: *p < 0.05, **p < 0.01, or ***p < 0.001; PLSD ANOVA test. Cell numbers in the dentate gyrus are mean ± SEM of the analysis of three animals per group. **(D)** The ratio of immature neurons (stage 5, BrdU^+^/Calretinin^+^/NeuN^+^) or of terminally differentiated neurons (stage 6, BrdU^+^/Calretinin^−^/NeuN^+^) to the total number of BrdU^+^ 23- to 28-day-old neurons was unchanged by fluoxetine treatment in the different genotypes, except in stage 6 wild-type neurons. Simple effects analysis: NS, *p* > 0.05, *p < 0.05, ***p < 0.001; Mann-Whitney U test.

**Figure 4:**
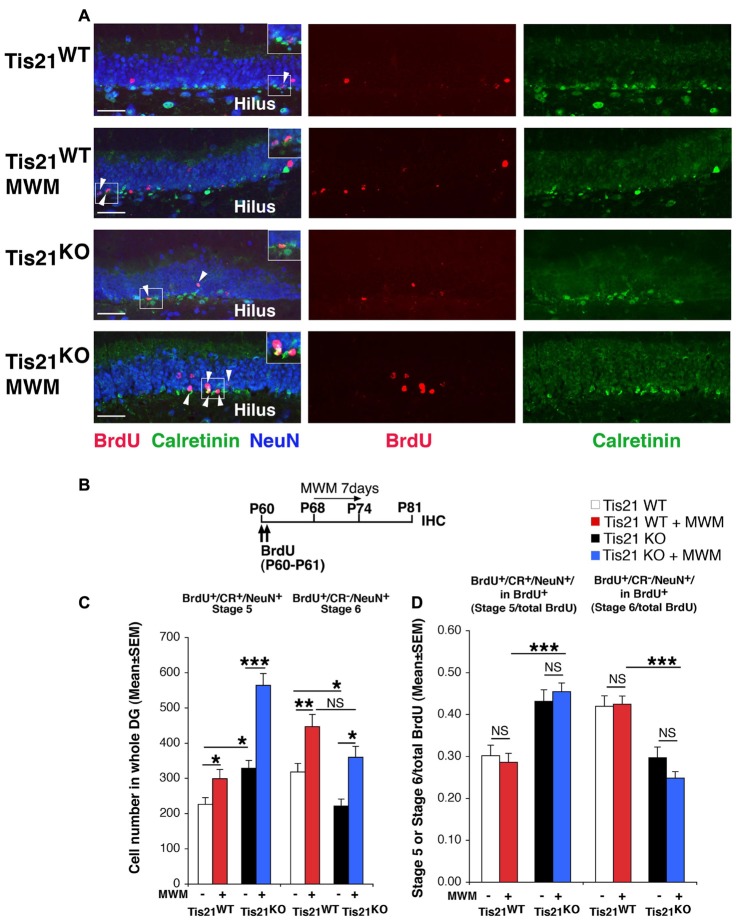
Spatial training in the Morris water maze (MWM) test increases the generation of early and late differentiated neurons in the adult dentate gyrus, but is unable to rescue the defect of terminal differentiation in Tis21 knockout neurons. **(A)** Representative confocal images (40× magnification) of the dentate gyrus of Tis21-null mice showing postmitotic immature neurons (stage 5, BrdU^+^/Calretinin^+^/NeuN^+^ cells; white arrowheads) and terminally differentiated neurons (stage 6, BrdU^+^/Calretinin^−^/NeuN^+^ cells); MWM-trained mice show a higher number of stage 5 and stage 6 neurons in the wild-type and in Tis21 knockout dentate gyrus. Scale bar, 50 μm. The white box area is shown with 1.5× digital magnification. **(B)** Timeline of the protocol followed to detect new 21-day-old differentiated neurons, with two daily injection of BrdU at days 0–1, followed by 7 days of MWM training (day 8–14) and analysis at day 21. **(C)** MWM training highly increases the new 21-day-old immature neurons (stage 5, BrdU^+^/Calretinin^+^/NeuN^+^) and the stage 6 differentiated neurons (BrdU^+^/Calretinin^−^/NeuN^+^) in wild-type as well as in Tis21 knockout dentate gyrus. Simple effects analysis: *p < 0.05, **p < 0.01, or ***p < 0.001; PLSD ANOVA test. Cell numbers in the dentate gyrus are mean ± SEM of the analysis of five animals per group. **(D)** The differentiation rate (calculated as ratio of stage 5 immature neurons or of stage 6 terminally differentiated neurons to the total number of BrdU^+^ 21-day-old neurons) did not change in the different genotypes. Simple effects analysis: NS, *p* > 0.05, ***p < 0.001; Mann-Whitney U test.

**Figure 5:**
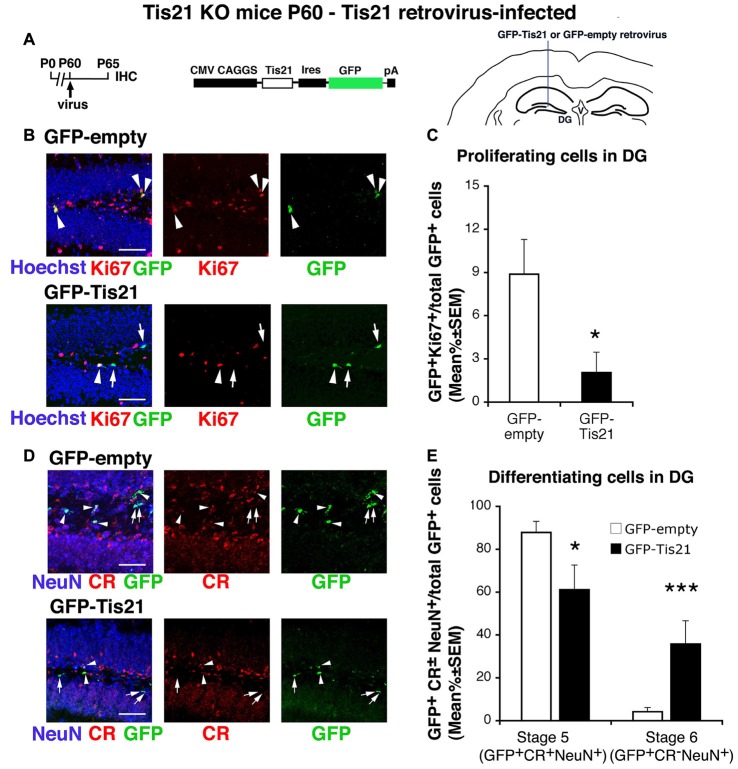
Tis21-retrovirus inhibits the deregulated proliferation of dentate gyrus progenitor cells and rescues the defective terminal differentiation of Tis21-null neurons. **(A)** Scheme of retrovirus infection timeline, structure, and injection area. **(B)** Representative confocal images (40× magnification) of coronal sections of the dentate gyrus, labeled with Hoechst 33258, Ki67 and with GFP, 5 days after infection with either GFP-Tis21 or GFP-empty retroviruses. Scale bars, 50 μm. The white arrowheads indicate cells positive for both GFP and Ki67, white arrows cells positive only for GFP. **(C)** Percentage ratio between GFP^+^Ki67^+^ cells and the total number of infected cells (GFP^+^), from the analysis of Tis21 knockout dentate gyrus infected with either GFP-Tis21 or GFP-empty virus. The percentage of dividing cells (Ki67^+^) is reduced by the Tis21 virus, relative to the empty virus infections. *p < 0.05 vs. cells infected with GFP-empty virus; Mann-Withney U test. **(D)** Representative confocal images of dentate gyrus cells triple labeled with Calretinin, NeuN and GFP, 5 days after infection with either GFP-Tis21 or GFP-empty retroviruses. Scale bars, 50 μm. White arrows indicate infected terminally differentiated neurons (GFP^+^Calretinin^−^NeuN^+^); arrowheads indicate infected stage 5 immature neurons (GFP^+^Calretinin^+^NeuN^+^). **(E)** Percentage ratio between stage 5 immature neurons (GFP^+^stage 5) or stage 6 terminally differentiated neurons (GFP^+^stage 6) and the total number of infected cells (GFP^+^), analyzed in Tis21 knockout dentate gyrus infected with either GFP-Tis21 or GFP-empty virus. The percentages of stage 5 and stage 6 neurons infected with empty virus are significantly decreased or increased, respectively, by the Tis21-expressing virus. *p < 0.05 or ***p < 0.001 vs. cells infected with GFP-empty virus; Mann-Withney U test. **(C,E)** Cell ratios in the dentate gyrus are mean% ± SEM of the analysis of three animals per group.

**Figure 6:**
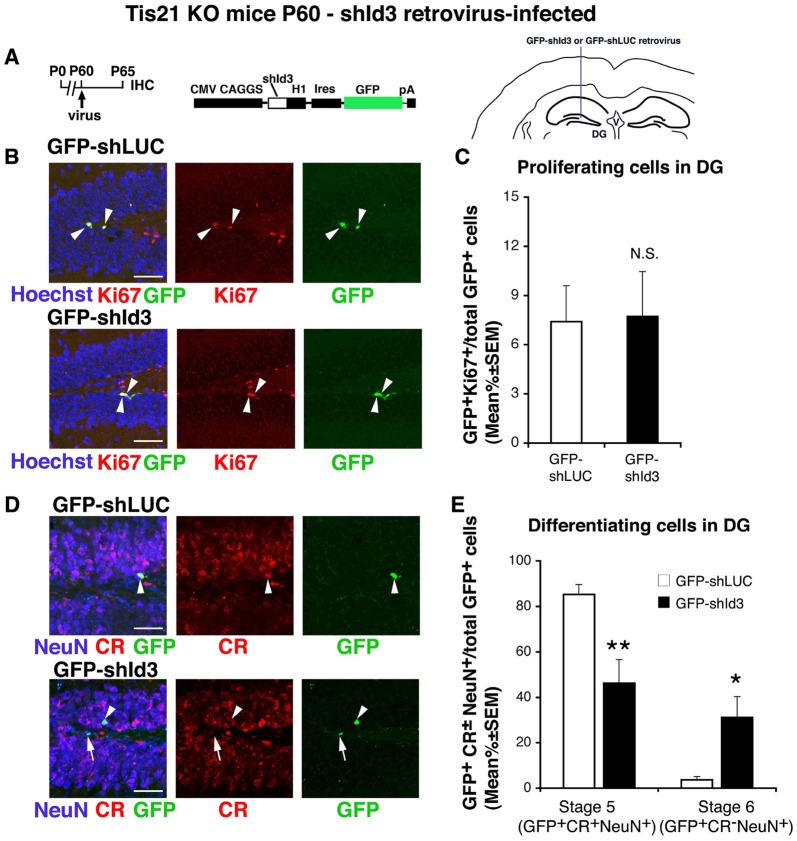
shId3-retrovirus rescues the defective terminal differentiation of Tis21-null neurons without effects on proliferating cells. **(A)** Retrovirus infection timeline, structure and injection area. **(B)** Representative confocal images (40× magnification) of coronal sections of the dentate gyrus, labeled with Hoechst 33258 and with Ki67 and GFP, 5 days after infection with either GFP-shId3 or GFP-shLUC (control) retroviruses. Scale bars, 50 μm. The white arrowheads indicate cells positive for both GFP and Ki67. **(C)** Percentage ratio between GFP^+^Ki67^+^ cells and the total number of infected cells (GFP^+^), in Tis21 knockout dentate gyrus infected with GFP-shId3 or GFP-shLUC virus. The ratio of dividing cells (Ki67^+^) is not changed by the shId3 virus, relative to the shLUC control virus infections. NS, *p* > 0.05 vs. cells infected with GFP-shLUCvirus; Mann-Withney U test. **(D)** Representative confocal images of dentate gyrus cells triple-labeled with Calretinin, NeuN and GFP, 5 days after infection with either GFP-shId3 or GFP-shLUC retroviruses. Scale bars, 50 μm. White arrows indicate infected terminally differentiated neurons (GFP^+^Calretinin^−^NeuN^+^); arrowheads indicate infected stage 5 immature neurons (GFP^+^Calretinin^+^NeuN^+^). **(E)** Percentage ratio between stage 5 immature neurons (GFP^+^stage 5) or stage 6 terminally differentiated neurons (GFP^+^stage 6) and the total number of infected cells (GFP^+^), analyzed in Tis21 knockout dentate gyrus infected with either GFP-shId3 or GFP-shLUC virus. The percentages of stage 5 neurons or stage 6 neurons observed in the shLUC virus infections are significantly decreased or increased, respectively, by the shId3 virus. *p < 0.05 or **p < 0.01 vs. cells infected with GFP-shLUC virus; Mann-Withney U test. **(C,E)** Cell ratios in the dentate gyrus are mean% ±SEM of the analysis of three animals per group.

**Figure 7:**
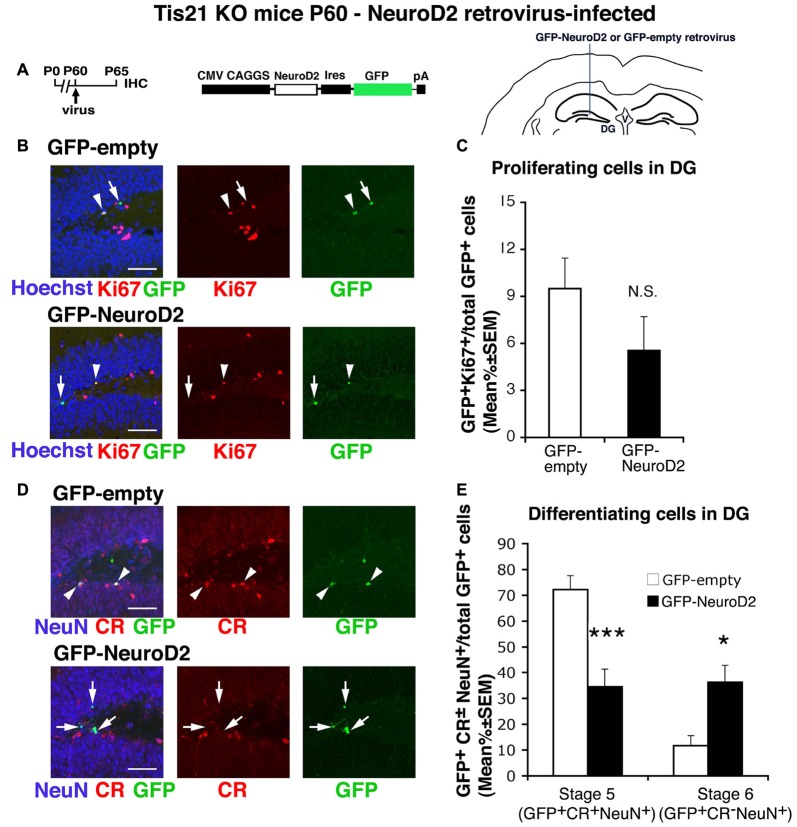
NeuroD2-retrovirus rescues the defective terminal differentiation of Tis21-null neurons without affecting the proliferation of progenitor cells. **(A)** Retrovirus infection timeline, structure and injection area. **(B)** Representative confocal images (40× magnification) of coronal sections of the dentate gyrus, labeled with Hoechst 33258, Ki67 and with GFP, 5 days after infection with either GFP-NeuroD2 or GFP-empty retroviruses. Scale bars, 50 μm. The white arrowheads indicate cells positive for both GFP and Ki67, white arrows cells positive only for GFP. **(C)** Percentage ratio between GFP^+^Ki67^+^ cells and the total number of infected cells (GFP^+^), in Tis21 knockout dentate gyrus infected with GFP-NeuroD2 or GFP-empty retroviruses. The percentage of dividing cells (Ki67^+^) is reduced, although not significantly, by the NeuroD2 virus, relative to control virus infections. NS, *p* > 0.05 vs. cells infected with GFP-empty retrovirus; Mann-Withney U test. **(D)** Representative confocal images of dentate gyrus cells triple-labeled with Calretinin, NeuN and GFP, 5 days after infection with either GFP-NeuroD2 or GFP-empty retroviruses. Scale bars, 50 μm. White arrows: infected terminally differentiated neurons (GFP^+^Calretinin^−^NeuN^+^); arrowheads: infected stage 5 immature neurons (GFP^+^Calretinin^+^NeuN^+^). **(E)** Percentage ratio of stage 5 immature neurons (GFP^+^stage 5) or stage 6 terminally differentiated neurons (GFP^+^stage 6) to the total number of infected cells (GFP^+^), analyzed in Tis21 knockout dentate gyrus infected with either GFP-NeuroD2 or GFP-empty retrovirus. The higher percentage of stage 5 and the lower percentage of stage 6 control-infected neurons are equalized by the NeuroD2 virus. *p < 0.05 or ***p < 0.001 vs. cells infected with GFP-empty virus; Mann-Withney U test. **(C,E)** Cell ratios in the dentate gyrus are mean% ± SEM of the analysis of three animals per group.

### Generation of Recombinant Viruses and Infection *In Vivo*

The retroviral vector pCAG-IRES-GFP, kindly provided by Dr. Chichung Lie (Institute of Developmental Genetics, Germany; Jessberger et al., 2008), was used to express only in dividing neural cells the cDNA of Tis21 (i.e., the murine sequence) and NeuroD2, as well as the shId3-190 RNA interfering sequence. The construct pCAG-IRES-GFP-Tis21 had been generated as previously described (Farioli-Vecchioli et al., 2014a). The construct pCAG-IRES-GFP-shId3 was generated by cloning in the SfiI-5′/PmeI-3′ sites of pCAG-IRES-GFP the whole 64-mer containing the 19-nucleotide siRNA from the pSUPER.retro-neo-GFP-shId3-190 vector, preceded by the H1 promoter and the TATA box sequence driving the transcription. pSUPER.retro-neo-GFP-shId3-190 was previously generated by us and the 19-nucleotide siRNA sequence specific to mouse Id3 was shown to efficiently reduce the Id3 mRNA levels (Farioli-Vecchioli et al., 2014a).

The same procedure was used to obtain the pCAG-IRES-GFP-shLUC retroviral vector, containing an sh-LUC control sequence specifically targeting the luciferase gene (Farioli-Vecchioli et al., 2014a). The pCAG-IRES-GFP-NeuroD2 sequence was obtained by cloning the full open reading frame of NeuroD2 mouse cDNA in the sites SfiI-5′/PmeI-3′ of pCAG-IRES-GFP. The shId3, shLUC and NeuroD2 sequences cloned into pCAG-IRES-GFP vector had been synthesized by MWG (Ebersberg, Germany) and were checked by DNA sequencing.

The different retroviruses, including pCAG-IRES-GFP-shLUC, were propagated as previously described (Farioli-Vecchioli et al., 2008). The concentrated virus solution (10^8^ pfu/ml) was infused (1.5 μl at 0.32 μl/min) by stereotaxic surgery into the right and left dentate gyrus of anesthetized P60 Tis21-null mice (anteroposterior = −2 mm from bregma; lateral = ±1.5 mm; ventral = 2.0 mm). Infected, GFP-positive cells were counted throughout the whole extent of the dentate gyrus.

All the retroviruses generated were amphotropic and replication-deficient. Their manipulation and stereotactic injection in mice were approved by the Italian Ministry of Health (authorizations RM/IC/Op2/06/008 and 04/2007) and performed using BSL-2 and ABSL-2 containments.

### Morris Water Maze

For the training in the MWM, the same protocol as in Farioli-Vecchioli et al. (2009) was used, with minor modifications. The training was carried out in a circular swimming pool of 1.3 m in diameter, filled with opaque (white) water kept at constant temperature (25 ± 1°C). The apparatus was located in a room containing prominent extra-maze cues. A hidden 15-cm-diameter platform was used. We measured the time employed by the mouse to reach the hidden platform (s). The training consisted of 28 trials (four trials per day, lasting a maximum of 60 s, with an inter-trial interval of 30 min), with the platform maintained in the same position. Mice were left for 15 s upon the platform at the end of each trial. In the event a mouse did not reach the platform within 60 s, it was gently directed to the platform by the experimenter and left for 15 s upon it (this occurred only sporadically on the first day). No probe test was performed. Only male mice (2 months of age; at least five per group) were used for the experiment. Mice behavior was recorded and analyzed offline by the EthoVision software (Noldus Information Technology, Wageningen, Netherlands).

### Statistical Analysis and Experimental Design

Two-way ANOVA was used to compare the effects in all experiments on wild-type and knockout mice of fluoxetine treatment (including cell cycle length) and of MWM training; individual between-group comparisons, where appropriate, were carried out by Fisher’s PLSD ANOVA *post hoc* test. The variance of data in the fluoxetine and MWM experiment that were calculated as ratio of differentiated neurons (stage 5 or stage 6) to the total number of bromodeoxyuridine^+^ (BrdU^+^) cells (Figures 3D, 4D), were instead analyzed with the Kruskall-Wallis test, which accounts for the assumption of non-normal distribution; individual between-group comparisons where then performed with the non parametric Mann-Whitney U test which does not require the assumption of normal distribution. Each experimental group analyzed was composed of at least three animals. Mann-Whitney U test was also used to analyze the percent values of retrovirally infected dentate gyrus cells, as the distribution of percent data may not comply with the assumption of a normal distribution. These analyses were performed using the StatView 5.0 software (SAS Institute, Cary, NC, USA). Supplementary Table S1 summarizes mice number, experimental design and statistical tests used for each experiment. Supplementary Table S2 summarizes two-way ANOVA and *post hoc* analyses.

Differences were considered statistically significant at *p* < 0.05. All data were expressed as mean values ± SEM.

## Results

We have previously demonstrated that ablation of Tis21 causes a selective impairment of terminal differentiation of stage 5 into stage 6 dentate gyrus neurons (Farioli-Vecchioli et al., 2009).

We sought to ascertain whether the genetic impairment of terminal differentiation could be rescued, at first by enhancing the proliferation of stem/progenitor cells, and hence the neurogenesis, by means of a pharmacological treatment—i.e., fluoxetine, which stimulates the serotonin pathway (Malberg et al., 2000)—or by a cognitive stimulus, the MWM (Epp et al., 2013). Alternatively, we analyzed the effect of genetic stimuli on differentiation.

### Fluoxetine Increases the Number of Dentate Gyrus Progenitor Cells in Wild-Type and Tis21 Knockout Mice

As a first step, we checked in our system the known ability of fluoxetine to induce the proliferation of progenitor cells of the dentate gyrus (Encinas et al., 2006; Crowther and Song, 2014; Bolijn and Lucassen, 2015). We treated 2-month-old mice with fluoxetine daily for 18 days, and then we analyzed the ongoing proliferation of dentate gyrus cells. We observed that the treatment with fluoxetine significantly increased the total number of proliferating stem and progenitor cells in the dentate gyrus (detected as Ki67^+^) in wild-type as well as in mutant mice, relative to the respective control-treated (with water) mice (Tis21^WT^ vs. Tis21^WT^ + FLX, 82% increase, *p* < 0.0001 PLSD test; Tis21^KO^ vs. Tis21^KO^ + FLX, 40% increase, *p* = 0.0003 PLSD test; two-way ANOVA, fluoxetine treatment effect *F*_(1,143)_ = 31.8, *p* < 0.0001; Figures 1A–C). Moreover, as expected, the knockout of Tis21 in itself led to increased proliferation of the total proliferating cells (Tis21^WT^ vs. Tis21^KO^, 55% increase, *p* = 0.005; Figures 1A,C). We further checked the increase of proliferation in the subpopulations of stem/progenitor cells. Type-1-2a cells were identified as nestin^+^DCX^−^, type-2b progenitor cells as nestin^+^ and DCX^+^, while type-3 cells as DCX^+^and nestin^−^ (Filippov et al., 2003; Kronenberg et al., 2003; Kempermann et al., 2004). We observed that in wild-type as well as in mutant mice the number of proliferating type-2b and type-3 progenitor cells was increased by fluoxetine treatment (type-2b, Ki67^+^/nestin^+^/DCX^+^ cells: Tis21^WT^ vs. Tis21^WT^ + FLX, 55% increase, *p* = 0.007; Tis21^KO^ vs. Tis21^KO^ + FLX, 37% increase, *p* = 0.016 PLSD test, two-way ANOVA, treatment effect *F*_(1,143)_ = 13.2, *p* = 0.0004; type-3, Ki67^+^/nestin^−^/DCX^+^ cells: Tis21^WT^ vs. Tis21^WT^ + FLX, 73% increase, *p* = 0.013 PLSD test; Tis21^KO^ vs. Tis21^KO^ + FLX, 43% increase, *p* = 0.002 PLSD test, two-way ANOVA, treatment effect *F*_(1,143)_ = 15.6, *p* = 0.0001; Figures 1A,D).

### Fluoxetine Shortens the S-Phase Length in Tis21 Knockout Dentate Gyrus Progenitor Cells

Then, we analyzed whether fluoxetine was in itself able to enhance the proliferation of progenitor cells of the dentate gyrus by modifying the length of the cell cycle. For this, we calculated the S phase length (Ts)—which is the main determinant of cell cycle duration—using a new accurate procedure (Brandt et al., 2012; Farioli-Vecchioli et al., 2014b; Figures 2A–D). We treated 39-day-old mice with fluoxetine daily for 21 days; this protocol was used in order to maximize the proliferative effects of fluoxetine. It turned out that fluoxetine treatment was unable to alter the Ts of the wild-type proliferating progenitor cells in 2-month-old mice, whereas Ts was significantly shortened in Tis21 knockout mice treated with fluoxetine, relative to either Tis21 knockout mice control-treated or wild-type control-treated (Tis21^KO^ vs. Tis21^KO^ + FLX, *p* = 0.02 PLSD test, two-way ANOVA, treatment effect *F*_(1,164)_ = 2.04, *p* = 0.15; Tis21^WT^ vs. Tis21^KO^ + FLX, *p* = 0.033 PLSD test; Figures 2A–C). We also checked whether fluoxetine affected the Ts of the subpopulation of progenitor cells at the threshold of differentiation, i.e., the type-3 neuroblasts, which express NeuroD1 (Roybon et al., 2009). These cells in fact, since they are at a pre-differentiation stage, may represent an interesting target for our analysis. However, no significant change of Ts induced by fluoxetine was observed in NeuroD1^+^ cells (two-way ANOVA, treatment effect *F*_(1,102)_ = 1.10, *p* = 0.29; Figure 2D). Moreover, the ablation of Tis21 in itself did not alter Ts in 2-month-old dentate gyrus progenitor cells, relative to wild-type mice (Figures 2C,D). However, considering that Tis21, in addition to the intrinsic pro-differentiative function, has antiproliferative properties (Farioli-Vecchioli et al., 2009), it seemed appropriate to test whether the deletion of Tis21 could in itself modify the cell cycle length at an earlier age —2 weeks— when the proliferation in the dentate gyrus is stronger (Gilley et al., 2011). We found that indeed the Ts of the total population of stem/progenitor cells in 14-day-old Tis21 knockout mice resulted significantly shorter than in wild-type mice (about 19% decrease, *p* = 0.0005; Figures 2E,F). Furthermore, a significant decrease was observed also for the Ts of the subpopulation of NeuroD1^+^ progenitor cells (21% decrease, *p* = 0.023; Figures 2E,G).

Taken together, these data indicated that: (i) fluoxetine enhances the proliferation of the adult wild-type dentate gyrus progenitor cells, but is able to accelerate the S phase only in mice ablated of Tis21 (probably because of a permissive condition consequent to the absence of the antiproliferative Tis21); and that (ii) the knockout of Tis21 in itself causes an acceleration of the S phase although detectable only in young mice.

### Fluoxetine Enhances the Generation of Early and Late Differentiated Neurons in the Dentate Gyrus, but Does Not Rescue the Defective Terminal Differentiation of Tis21 Knockout Neurons

Having ascertained that fluoxetine exerts a powerful proliferative effect in mice of about 2-months of age on Tis21 knockout and wild-type dentate gyrus progenitor cells, we studied the effect of fluoxetine treatment on their terminal differentiation.

P60 mice were treated with fluoxetine for 18 days and injected with BrdU daily for the last 5 days of fluoxetine treatment. Dentate gyrus cells were analzyed at the end of the treatment, in this way monitoring essentially the proliferative effect of fluoxetine on progenitor cells. We observed that, although fluoxetine induced in both wild-type and knockout mice an increase of the number of immature and terminally differentiated neurons, fluoxetine was not able to restore their rate of differentiation (measured over the total number of BrdU^+^ cells) in mutant mice (data not shown and Supplementary Table S1).

Thus, we modified the protocol of treatment by adding at the end an off treatment period of 10 days, in order to allow the differentiation of the newly generated progenitor cells, and to better assess whether fluoxetine is able to accelerate the differentiation of progenitor cells in mutant mice (Supplementary Figures S1A–C).

We observed that fluoxetine significantly increased in both wild-type and knockout mice the number of stage 5 (BrdU^+^/Calretinin^+^/NeuN^+^, hereafter named as BrdU^+^/stage 5, Tis21^WT^ vs. Tis21^WT^ + FLX, *p* = 0.02 PLSD test; Tis21^KO^ vs. Tis21^KO^ + FLX, *p* = 0.009 PLSD test, two-way ANOVA, treatment effect *F*_(1,121)_ = 11.74, *p* = 0.0008; Supplementary Figure S1C) and stage 6 neurons (BrdU^+^/Calretinin^−^/NeuN^+^, hereafter named as BrdU^+^/stage 6, Tis21^WT^ vs. Tis21^WT^ + FLX, *p* = 0.008 PLSD test; Tis21^KO^ vs. Tis21^KO^ + FLX, *p* = 0.005 PLSD test, two-way ANOVA, treatment effect *F*_(1,121)_ = 15.23, *p* = 0.0002; Supplementary Figure S1C).

Notably, while the rate of differentiation after fluoxetine, measured as the ratio of terminally differentiated neurons to the total number of BrdU^+^ 10- to 15-day-old neurons, was restored to normal values in stage 5 neurons, the rate of stage 6 neurons remained lower in mutant mice relative to wild-type, indicating that no rescue of the defect of terminal differentiation occurred (BrdU^+^/stage 6 in total BrdU^+^: Tis21^WT^ + FLX vs. Tis21^KO^ + FLX, *p* < 0.0001, Mann-Whitney U test, Kruskall-Wallis (d.f. 3) *H* = 45.42, *p* < 0.0001; Supplementary Figure S1D).

Next, we decided to modify the protocol by anticipating BrdU injections at the beginning of the treatment, in order to detect the initial amplification of the different cohorts of neurons throughout the process of maturation during the treatment with fluoxetine, which is also endowed with a prodifferentiative action (Wang et al., 2008). We left the off treatment period of 10 days at the end, to allow the complete differentiation of the newly generated progenitor cells. Such protocol would highlight the effect of fluoxetine treatment not only on the proliferation and differentiation but also on the survival of progenitor cells (Encinas et al., 2006).

As a preliminary test, we checked whether the new conditions, with five daily injections of BrdU from the first day of treatment, allow to detect the proliferative effect of fluoxetine (Supplementary Figures S2A–C). In fact, a previous study indicated that an increase of BrdU^+^ cells by fluoxetine was detected between 5 days and 14 days of treatment, after, however, a single injection of BrdU before treatment (Malberg et al., 2000). We observed that BrdU^+^ cells are significantly increased by fluoxetine treatment for 7 days (BrdU^+^, Tis21^WT^ vs. Tis21^WT^ + FLX, one-way ANOVA *F*_(1,70)_ = 4.6, *p* = 0.035; Supplementary Figure S2C), plausibly before a fluoxetine-dependent survival action may take place (Caiaffo et al., 2016). Therefore, our protocol appears to be suited to detect the effect of fluoxetine on both proliferation and differentiation of progenitor cells, with also a prosurvival component that certainly plays a part during the treatment.

Thus, we induced the process of neurogenesis by treating P60 mice with fluoxetine for 18 days, followed by a period of 10 days in the absence of treatment, to allow the differentiation of the newly generated progenitor cells. New neurons were labeled by five daily injections of BrdU performed during the first 5 days of fluoxetine treatment (Figures 3A,B; see scheme 3B). This protocol, from Wang et al. (2008) with modifications, allows detection of new 23- to 28-day-old differentiated neurons, generated with or without the neurogenic stimulus of fluoxetine.

We observed an increase of immature stage 5 neurons in Tis21 knockout mice, relative to wild-type (BrdU^+^/stage 5 neurons, *p* = 0.028 PLSD test; Figures 3A,C) and a decrease of terminally differentiated stage 6 neurons (BrdU^+^/stage 6 neurons, *p* = 0.03; Figures 3A,C), as expected. Fluoxetine significantly increased in wild-type and in mutant mice the number of differentiated neurons, either immature (BrdU^+^/stage 5, Tis21^WT^ vs. Tis21^WT^ + FLX, *p* = 0.007 PLSD test; Tis21^KO^ vs. Tis21^KO^ + FLX, *p* = 0.0013 PLSD test, two-way ANOVA, treatment effect *F*_(1,80)_ = 18, 466, *p* < 0.0001; Figure 3C), or terminally differentiated (BrdU^+^/stage 6, Tis21^WT^ vs. Tis21^WT^ + FLX, *p* < 0.0001 PLSD test; Tis21^KO^ vs. Tis21^KO^ + FLX, *p* = 0.0004 PLSD test, two-way ANOVA, treatment effect *F*_(1,79)_ = 72.355, *p* < 0.0001; Figure 3C). The increase of 28-day-old neurons confirms that the cell cycle acceleration of progenitor cells induced by fluoxetine enhances the generation of viable neurons. Fluoxetine treatment, however, was unable to functionally counteract the impairment of differentiation occurring in Tis21 knockout mice, as the number of terminally differentiated neurons (BrdU^+^/stage 6) observed in mutant mice after fluoxetine treatment remained significantly lower than in wild-type mice treated with fluoxetine (Tis21^KO^ + FLX vs. Tis21^WT^ + FLX, *p* < 0.0001 PLSD test; Figure 3C).

Moreover, if we visualize in Figure 3D the rate of differentiation by analyzing the ratio of terminally differentiated neurons (BrdU^+^/stage 6) to the total number of BrdU^+^ 23- to 28-day-old neurons, this was slightly but significantly changed by fluoxetine in wild-type, indicating a mild pro-differentiative effect by fluoxetine. However, again, no change by fluoxetine occurred in the ratio of immature or terminally differentiated neurons of mutant mice (BrdU^+^/stage 5 in total BrdU^+^: Tis21^WT^ vs. Tis21^WT^ + FLX, *p* = 0.14 or Tis21^KO^ vs. Tis21^KO^ + FLX, *p* = 0.09 Mann-Whitney U test, Kruskall-Wallis (d.f. 3) *H* = 38.92, *p* < 0.0001; BrdU^+^/stage 6 in total BrdU^+^: Tis21^WT^ vs. Tis21^WT^ + FLX, *p* = 0.017 or Tis21^KO^ vs. Tis21^KO^ + FLX, *p* = 0.30 Mann-Whitney U test, Kruskall-Wallis (d.f. 3) *H* = 30.96, *p* < 0.0001; Figure 3D). In fact, the pattern of increase of stage 5 neurons and the decrease of stage 6 neurons elicited by Tis21 knockout, relative to wild-type mice, remained the same also after fluoxetine treatment (BrdU^+^/stage 5 in total BrdU^+^: Tis21^WT^ + FLX vs. Tis21^KO^ + FLX, *p* < 0.0001, Mann-Whitney U test; BrdU^+^/stage 6 in total BrdU^+^: Tis21^WT^ + FLX vs. Tis21^KO^ + FLX, *p* < 0.0001, Mann-Whitney U test; Figure 3D).

As a whole, this indicates that the defect of terminal differentiation of stage 5 into stage 6 neurons in Tis21 knockout mice is refractory to rescue by the proliferative and pro-differentiative neurogenic stimulus of fluoxetine, and suggests that this process is separate from the expansion of progenitor cells.

### Training in the Morris Water Maze Enhances Neurogenesis in the Dentate Gyrus, but Fails to Rescue the Defective Terminal Differentiation of Tis21 Knockout Neurons

As a second step, we tested whether a different neurogenic stimulus —namely, spatial learning in the MWM— could rescue the differentiation defect of Tis21 knockout dentate gyrus. It has in fact been demonstrated that spatial learning activates neurogenesis and promotes the survival and incorporation of new neurons into the dentate gyrus (Gould et al., 1999). The neurogenic effect of spatial learning appears more pronounced in 1-week-old neurons, as determined by BrdU birth-dating (Epp et al., 2013). We decided to highlight the process of neuron differentiation, by analyzing the new neurons generated 21 days after two daily injections of BrdU, whereas the MWM training was performed from day 8 to day 14, when the proliferative phase was almost concluded (Figures 4A,B, see scheme 4B). This timeline was chosen following, with modifications, published protocols (Dupret et al., 2007; Epp et al., 2007). The experiments were performed in 2-month-old mice.

The MWM was performed as described in Farioli-Vecchioli et al. (2009) with minor modifications. In this task, mice learn across daily sessions to find a hidden escape platform using extra-maze visual cues. Tis21^KO^ and Tis21^WT^ mice performed equally in the task (Supplementary Figure S3). MWM training elicited in the dentate gyrus of both wild-type and Tis21 knockout mice a significant increase of immature neurons (BrdU^+^/stage 5, Tis21^WT^ vs. Tis21^WT^ + MWM, *p* = 0.042; Tis21^KO^ vs. Tis21^KO^ + MWM, *p* < 0.0001 PLSD test, two-way ANOVA, MWM effect *F*_(1,270)_ = 27.17, *p* < 0.0001; Figure 4C), as well as of terminally differentiated neurons (BrdU^+^/stage 6, Tis21^WT^ vs. Tis21^WT^ + MWM, *p* < 0.002 PLSD test; Tis21^KO^ vs. Tis21^KO^ + MWM, *p* = 0.011 PLSD test, two-way ANOVA, MWM effect *F*_(1,270)_ = 15.35, *p* = 0.0001; Figure 4C). Notably, after MWM training the difference between the number of wild-type and Tis21 knockout terminally differentiated neurons was reduced to a non-significant level, suggesting that some rescue of terminal differentiation occurred (BrdU^+^/stage 6, Tis21^WT^ + MWM vs. Tis21^KO^ + MWM, *p* = 0.070 PLSD test; Figure 4C). Nevertheless, when we analyzed the ratio of stage 5 or 6 neurons to the total number of BrdU^+^ neurons, no change was exerted by MWM training (BrdU^+^/stage 5 in total BrdU^+^: Tis21^WT^ vs. Tis21^WT^ + MWM, *p* = 0.87 or Tis21^KO^ vs. Tis21^KO^ + MWM, *p* = 0.37 Mann-Whitney U test, Kruskall-Wallis (d.f. 3) *H* = 30.41, *p* < 0.0001; BrdU^+^/stage 6 in total BrdU^+^: Tis21^WT^ vs. Tis21^WT^ + MWM, *p* = 0.68 or Tis21^KO^ vs. Tis21^KO^ + MWM, *p* = 0.57 Mann-Whitney U test, Kruskall-Wallis (d.f. 3) *H* = 36.17, *p* < 0.0001; Figure 4D). Hence, the Tis21 knockout-induced pattern of increase of stage 5 neurons and decrease of stage 6 neurons, relative to wild-type mice, remained the same also after MWM training, indicating that the defect in the differentiation rate of Tis21 knockout neurons was not rescued (BrdU^+^/stage 5 in total BrdU^+^: Tis21^WT^ + MWM vs. Tis21^KO^ + MWM, *p* < 0.0001, Mann-Whitney U test; BrdU^+^/stage 6 in total BrdU^+^: Tis21^WT^ + MWM vs. Tis21^KO^ + MWM, *p* < 0.0001, Mann-Whitney U test; Figure 4D).

### The Impairment of Differentiation in Tis21-Null Progenitor Cells of the Dentate Gyrus Is Rescued by Id3 Silencing and by NeuroD2

As a way to assess the specificity of impairment of the differentiation observed in Tis21 knockout mice, we sought to rescue it by injecting in the dentate gyrus retroviruses expressing (or silencing) either Tis21 (Figure 5) or genes regulating neural differentiation at specific steps, namely, Id3 (Figure 6) and NeuroD2 (Figure 7). The gene Id3, which negatively regulates neural differentiation (Lyden et al., 1999; Andres-Barquin et al., 2000; Yokota, 2001), has been previously shown to be strongly repressed by Tis21 in dentate gyrus neurons (Farioli-Vecchioli et al., 2009); whereas NeuroD2 is involved in the process of dentate gyrus development and survival (Olson et al., 2001) and is amongst the bHLH genes target of the inhibitory activity of Id3.

The infection of the dentate gyrus of Tis21-null adult mice (at P60) with a retrovirus expressing Tis21 and GFP (pCAG-IRES-GFP-Tis21; Figures 5A–E) occurred only in proliferating progenitors; however, the analysis of infected cells (GFP^+^) was performed 5 days after infection, hence cells were detected that either continued to proliferate or had differentiated. The pCAG-IRES-GFP-Tis21 virus effectively inhibited the proliferation of dividing progenitors (GFP-Tis21^+^Ki67^+^/total GFP-Tis21^+^ vs. GFP-empty^+^Ki67^+^/total GFP-empty^+^ *p* = 0.015, Mann Whitney U test, used hereafter for virus infection analyses; Figures 5A,C); in parallel, the high percentage of immature stage 5 neurons and the low percentage of terminally differentiated stage 6 neurons present in Tis21-null dentate gyrus was reversed (stage 5: GFP-Tis21^+^stage 5/total GFP-Tis21^+^ vs. GFP-empty^+^stage 5/total GFP-empty^+^ *p* = 0.037; stage 6: GFP-Tis21^+^stage 6/total GFP-Tis21^+^ vs. GFP-empty^+^stage 6/total GFP-empty^+^ *p* = 0.001; Figures 5D,E). These data clearly indicate that the defect of differentiation of dentate gyrus progenitor cells is specifically dependent on the loss of Tis21.

Given that non-specific stimuli triggering the whole neurogenic machinery, either by activation of the serotoninergic pathway, or promoting the process of differentiation through cognitive training, did not rescue the defect of terminal differentiation, we now tested the specific silencing of Id3. In fact Id3 is an inhibitor of the proneural bHLH genes (Lyden et al., 1999; Yokota, 2001), and is negatively regulated at transcriptional level by Tis21 in neural cells (Farioli-Vecchioli et al., 2009).

Thus, we infected the dentate gyrus cells of Tis21-null mice with a retrovirus expressing GFP and an shRNA targeting specifically Id3 (GFP-shId3), which had been previously generated and checked for its efficiency in silencing Id3 expression (Farioli-Vecchioli et al., 2009; Figures 6A–E). We observed that the percentage of dividing progenitors observed in Tis21-null mice was not affected by the infection with the GFP-shId3 retrovirus, as compared to a control retrovirus expressing an shRNA targeting luciferase (GFP-shLUC, Farioli-Vecchioli et al., 2014a; GFP-shId3^+^Ki67^+^/total GFP-shId3^+^ vs. GFP-shLUC^+^Ki67^+^/total GFP-shLUC^+^ *p* = 0.35; Figures 6A,C). In contrast, the percent accumulation of immature neurons and the lower percent ratio of terminally differentiated neurons occurring in Tis21-null dentate gyrus were reversed by GFP-shId3 retrovirus (stage 5: GFP-shId3^+^stage 5/total GFP-shId3^+^ vs. GFP-shLUC^+^stage 5/total GFP-shLUC^+^ *p* = 0.006; stage 6: GFP-shId3^+^stage 6/total GFP-shId3^+^ vs. GFP-shLUC^+^stage 6/total GFP-shLUC^+^ *p* = 0.043; Figures 6D,E). Of note, the measurement of stage 5/6-infected cells (i.e., GFP^+^) was performed in all infection experiments as ratio to the total number of GFP^+^ cells, and is therefore a percentage analysis analogous to that used to measure FLX-treated progenitor cells above background in Figure 3D. Thus, the silencing of Id3 expression specifically rescues the Tis21 knockout-dependent defect of differentiation of dentate gyrus progenitor cells, without effect on their proliferation. This reveals that Id3 is a specific regulator of the terminal differentiation of dentate gyrus progenitor cells, where it acts under control of Tis21 (Farioli-Vecchioli et al., 2009), and that proliferation and differentiation in this system are genetically dissociable.

We further tested whether the proneural gene NeuroD2 could specifically rescue the defect of terminal differentiation of Tis21-null neurons. This choice was based on the fact that NeuroD2 has been shown to induce neural differentiation in embryonic stem cells (Sugimoto et al., 2009) and to be selectively expressed in stage 6 dentate gyrus neurons, i.e., with Tis21 (Roybon et al., 2009; Attardo et al., 2010).

Thus, we generated a retrovirus expressing GFP and the NeuroD2 coding region (GFP-NeuroD2) and used it to infect the dentate gyrus cells of Tis21-null mice. We noticed a manifest decrease, albeit below significance, of the proliferating stem/progenitor cells expressing the NeuroD2 virus, relative to control-infected cells (GFP-NeuroD2^+^Ki67^+^/total GFP-NeuroD2^+^ vs. GFP-empty^+^Ki67^+^/total GFP-empty^+^ *p* = 0.15; Figures 7A,C). In contrast, the higher percentage of stage 5 immature neurons and the lower percentage of terminally differentiated neurons occurring in Tis21-null dentate gyrus were overturned in cells infected by the NeuroD2 retrovirus (stage 5: GFP-NeuroD2^+^stage 5/total GFP-NeuroD2^+^ vs. GFP-empty^+^stage 5/total GFP-empty^+^ *p* = 0.0004; stage 6: GFP-NeuroD2^+^stage 6/total GFP-NeuroD2^+^ vs. GFP-empty^+^stage 6/total GFP-empty^+^ *p* = 0.044; Figures 7D,E). This indicates that NeuroD2, similarly to Id3, is a specific regulator of the terminal differentiation of dentate gyrus progenitor cells, able to rescue the defect of differentiation in Tis21-null dentate gyrus neurons.

## Discussion

We have previously shown that the timing of differentiation is crucial for the correct maturation and function of the hippocampal neuron. In fact we have observed that the acceleration of the process of differentiation, exerted by conditionally overexpressing in dentate gyrus progenitor cells, the pro-differentiative gene Tis21 activated in a transgenic mouse, drastically impairs the functionality of the neuron to encode and retrieve memories. Such impairment is more pronounced than that caused by the deletion of new neurons, and is probably consequent to the dominant negative effect of recruiting in memory circuits a functionally defective new neuron (Farioli-Vecchioli et al., 2008; Tirone et al., 2013).

Here we observe that in a neuron with a genetic impairment of terminal differentiation, dependent on the ablation of Tis21, a neurogenic stimulus triggering a very strong proliferation of progenitor cells such as that induced by fluoxetine, although able to accelerate the cell cycle rate of Tis21-knockout dentate gyrus progenitor cells, is not sufficient to enhance the rate of differentiation. Our aim was to test whether it was possible to rescue the defect of differentiation by increasing the pool of progenitor cells committed to differentiation, using a neurogenic stimulus that is mainly proliferative, like fluoxetine, but at the same time able to accelerate the maturation of immature DCX^+^ cells (Wang et al., 2008) and also to promote the survival (Encinas et al., 2006). We also considered that stage 5 cells, whose differentiation is impaired in Tis21 knockout dentate gyrus, are immature DCX^+^ cells. Notably, recently Brandt et al. (2012) showed that progenitor cells type-2b/3 (NeuroD1^+^) divide faster than earlier progenitor cells, suggesting that cells committed to amplification progress faster toward differentiation. We reasoned that a neurogenic stimulus may trigger such activation. These concepts are compatible also with the notion that cell cycle-dependent mechanisms modulate the activation of differentiation genes (Dalton, 2015). The idea to test the application of a strong external neurogenic stimulus came also from the fact that the increase of proliferation resulting from the deletion of Tis21 is temporally separated from the impairment of terminal differentiation, given that Tis21 is not expressed in stage 5 cells.

Fluoxetine increases the proliferation of progenitor cells, probably by increasing the serotonin (5HT) available to bind the 5HT3,4,6,7 receptors present in the hippocampus, and/or by inhibiting p21 expression (Encinas et al., 2006; Pechnick et al., 2011; Bolijn and Lucassen, 2015). Remarkably, despite the strong neurogenic action, we did not uncover any effect of fluoxetine on the length of the cycle of wild-type stem and progenitor cells, unlike what we have observed for another neurogenic stimulus, i.e., physical exercise (Farioli-Vecchioli et al., 2014b). This indicates that a neurogenic stimulus can induce neural proliferation without necessarily altering cell cycle length. Nevertheless, a significant acceleration of the S phase is exerted by fluoxetine in the progenitor cells lacking Tis21, probably as a consequence of the missing control of cell cycle normally exerted by Tis21. Notably, fluoxetine appears to be endowed also with a mild intrinsic pro-differentiative effect on new wild-type neurons, as in our treatment we observe an increase of the differentiation rate (ratio of 28-day-old neurons to the total number of new neurons generated, Figure 3D). A direct pro-differentiative effect of fluoxetine in dentate gyrus neurons had not been observed by Encinas et al. (2006), while it has been reported by Wang et al. (2008), also in line with the finding that fluoxetine increases BDNF expression in the bodies of dentate gyrus cells, where it has a strong influence on their maturation (Molteni et al., 2006; Waterhouse et al., 2012).

We adopted different protocols of treatment with fluoxetine, aimed to highlight its proliferative effect on dentate gyrus progenitor cells either alone or together with the pro-differentiation and survival effects (see Supplementary Table S1). In fact, we observe that while the neurogenic stimulus by fluoxetine highly increases the absolute number of terminally differentiated neurons (stage 6), relative to control-treated wild-type or Tis21-null dentate gyrus, that stimulus is unable to change the ratio of Tis21-null stage 6 neurons to the background production of new neurons. This indicates that changes in proliferation and/or in cell cycle length—as that observed in Tis21 knockout progenitor cells after fluoxetine treatment—do not interfere with the rate of differentiation once a genetic impairment of the process is present. No significant influence on the Tis21 differentiation defect is observed even when the proliferative stimuli are coupled to paradigms favoring the differentiation and survival by fluoxetine (Figure 3). However, in an experiment highlighting both the proliferative and differentiative effects of fluoxetine (Supplementary Figure S1), the ratio of Tis21-null stage 5 neurons to the background production of new neurons is rescued to almost normal values. Thus, the increased amount of stage 5 neurons terminally differentiates into stage 6, although this transition rate remains slower than in normal neurons. This defect of terminal differentiation is not rescued even by a neurogenic cognitive stimulus, such as the training in a hippocampus-dependent spatial memory task (Figure 4). We exploited this stimulus, which induces the generation of new neurons in the dentate gyrus, with a protocol aimed to specifically monitor the new neurons differentiated following the stimulus. As a whole, this suggests that the efficiency of the process of terminal differentiation is primarily dependent on the genetic control of that specific step and is rather independent from stimuli that trigger mainly the cellular division, despite these are likely to impact on cohorts of genes involved not only in the proliferative process but also in the first steps of differentiation (Julian et al., 2016). Apparently, the rate of differentiation of a progenitor cell that has attained the early postmitotic condition becomes independent from the pre-existing proliferative control, but the time required to attain such a condition may vary depending on the proliferative state and on the differentiative condition downstream.

Thus, it is not sufficient to merely enhance the amplification of neural stem cells in order to generate new neurons, as shown, for instance, by Artegiani et al. (2011) through induction of cyclin D1, unless the cell is in a mode permissive for differentiation.

This view would also agree with the observation made in spinal cord progenitor cells that forcing their proliferation, by overexpression of cyclin D, does not alter the neuronal fate, indicating that neuronal specification and differentiation are controlled independently of cell cycle exit (Lobjois et al., 2008).

In contrast, the specific upregulation in Tis21-null proliferating dentate gyrus progenitor cells of NeuroD2, or the silencing of Id3 by retroviral infection, determine a specific rescue of terminal differentiation of dentate gyrus neurons, further indicating that amplification and differentiation of neural progenitor cells are processes genetically separated.

This rescue also represents the first demonstration that NeuroD2 or the silencing of Id3 are able to activate the differentiation of dentate gyrus neurons.

We selected NeuroD2 as it is normally expressed in terminally differentiated (stage 6) dentate gyrus neurons (Roybon et al., 2009), concomitantly with Tis21 (Attardo et al., 2010). Thus, we asked whether NeuroD2 could substitute for Tis21. It has been previously shown that the induction of the expression of NeuroD2 in Xenopus embryos and P19 embryonal carcinoma cell line, as well as in embryonic stem cells, is sufficient to induce neural differentiation (McCormick et al., 1996; Farah et al., 2000; Sugimoto et al., 2009). Moreover, NeuroD2 is expressed in the hippocampus where it is required for survival of hippocampal neurons (Olson et al., 2001) and where it inhibits REST, involved in differentiation (Ravanpay et al., 2010; Kim et al., 2015). Interestingly, recently Richetin et al. (2015) did not observe an increase in the number of differentiating neurons in the adult dentate gyrus infected with a retrovirus expressing NeuroD2, whereas an increase was observed for NeuroD1-infected cells. It is possible that, in a system where the NeuroD1 pro-differentiative action prevails, only specific conditions—such as those occurring in the differentiation-defective Tis21 knockout system—can reveal the stage-specific pro-differentiative action of NeuroD2. Presently we do not know whether Tis21 regulates NeuroD2, although one possibility is that this occurs indirectly through Id3. Future studies will be necessary to assess this point and whether also other mechanisms or molecular partners are able to rescue the Tis21-dependent defect of terminal differentiation.

Concerning Id3, the Id proteins have a helix-loop-helix (HLH) dimerization domain but lack the DNA-binding domain and sequester E proteins, thus preventing their association to proneural basic HLH transcription factors, such as NeuroD1 or NeuroD2, in this way inactivating them (Lyden et al., 1999; Andres-Barquin et al., 2000; Yokota, 2001). In neural crest cells, Id3 has been shown to positively control the proliferation of neural cells, besides inhibiting their differentiation (Kee and Bronner-Fraser, 2005).

We have previously shown that in dentate gyrus Id3 is inhibited by Tis21, as the knockout of Tis21 greatly induces Id3 expression in that region (Farioli-Vecchioli et al., 2009). This increase of Id3 is determined by the binding of the Tis21 protein to the Id3 promoter, and is associated with a significant decrease of Calbindin, marker of the terminally differentiated dentate gyrus neurons (Farioli-Vecchioli et al., 2009). Since we show here that the silencing of Id3 rescues the defective differentiation of progenitor cells of the dentate gyrus, this indicates that Id3 controls their terminal differentiation pathway, and that Id3 is effector of Tis21, being also transcriptionally regulated by Tis21 (Farioli-Vecchioli et al., 2009). Furthermore, it is worth noting that Id3 controls the differentiation also of neuroblasts of the subventricular zone, since Id3 silencing rescues the defective differentiation observed in Tis21-null neurospheres (Farioli-Vecchioli et al., 2014a).

Altogether, our demonstration that the overexpression of NeuroD2 or the silencing of Id3 is able to restore a condition of defective differentiation is critical as it points to the possibility to use these targets to treat neurodegenerative diseases where defects of terminal differentiation of new dentate gyrus neurons occur, such as in Alzheimer’s disease, as we have previously proposed (Tirone et al., 2013). Accordingly, it has been recently demonstrated that by enhancing the differentiation of dentate gyrus progenitor cells through NeuroD1 overexpression it is possible to counteract the memory defects in a mouse model of Alzheimer’s disease (Richetin et al., 2015). Moreover, the selective expression of NeuroD2 in stage 6 neurons would suggest a specificity in targeting terminal differentiation higher than that of NeuroD1, which is highly expressed at an earlier stage, in immature neurons (Pleasure et al., 2000). These data also suggest that NeuroD2 could be no less effective than NeuroD1 in restoring the differentiative function in a neurodegenerative condition.

## Author Contributions

LM, MCeccarelli, DS and FT designed the experiments and interpreted the data; LM, MCeccarelli, RG, GD, SF-V, MCostanzi and DS carried out the experimental work; LM and FT wrote the article; LM, MCeccarelli, VC and FT are responsible for accuracy and integrity of any part of the work.

## Conflict of Interest Statement

The authors declare that the research was conducted in the absence of any commercial or financial relationships that could be construed as a potential conflict of interest.

## Supplementary Material

FIGURE S1 Fluoxetine, administered with a protocol highlighting proliferation and differentiation, rescues stage 5 but not stage 6 neurons defective for terminal differentiation in Tis21 knockout mice. **(A)** Representative confocal images (40×) showing stage 5 (BrdU^+^/Calretinin^+^/NeuN^+^; white arrowheads), and stage 6 neurons, (BrdU^+^/Calretinin^−^/NeuN^+^), treated as described in **(B)**. Scale bar, 100 μm. **(B)** Two-month-old mice received five daily injection of BrdU at the end of the fluoxetine treatment, followed by 10 days off treatment to allow differentiation. **(C)** Quantification of the number of new 15-day-old cells shows increase in Tis21-null dentate gyrus of stage 5 immature neurons and decrease of terminally differentiated stage 6 neurons, relative to wild-type. Fluoxetine increases both stage 5 and stage 6 neurons in wild-type and Tis21 knockout dentate gyrus. Simple effects analysis: **p* < 0.05, ***p* < 0.01, or ****p* < 0.001; PLSD ANOVA test. Cell numbers in the dentate gyrus are mean ± SEM of the analysis of four animals per group. **(D)** The ratio of stage 5 neurons to the total number of BrdU^+^ cells was restored by fluoxetine in mutant cells to the values of wild-type cells; nevertheless, no rescue was observed for fluoxetine-treated stage 6 mutant neurons that remained lower than control. Simple effects analysis: NS, *p* > 0.05, ****p* < 0.001; Mann-Whitney U test.

FIGURE S2 Fluoxetine treatment induces after 7 days a significant increase of the number of dentate gyrus progenitor cells in adult wild-type mice, as detected following multiple BrdU injections. **(A)** Representative confocal images (40× magnification) showing proliferating dentate gyrus progenitor cells, labeled by BrdU (red), in mice treated as described in **(B)**. Dotted lines delimit the outer boundary of the granule cell layer. Scale bar, 100 μm. **(B)** Two-month-old mice received five daily injection of BrdU at the beginning of the fluoxetine treatment, which lasted 7 days. **(C)** Quantification of total proliferating adult progenitor cells in wild-type dentate gyrus, measured as BrdU^+^ cells. Cell numbers in the dentate gyrus are mean ±SEM of the analysis of five animals per group. **p* < 0.05; PLSD ANOVA test.

FIGURE S3 Morris water maze (MWM) escape latency in wild-type and mutant mice. The MWM was performed as in Farioli-Vecchioli et al. (2009) with minor modifications. In this task, mice learn across daily sessions to find a hidden escape platform using extra-maze visual cues. Tis21^KO^ and Tis21^WT^ mice (both groups, *n* = 5) performed equally in the task. Statistical analysis (repeated measures ANOVA) showed a significant effect of training (*F*_(6,48)_ = 10.26; *p* < 0.0001), no significant effect of genotype (*F*_(1,8)_ = 1.87; *p* = 0.21) and no significant genotype × training interaction (*F*_(6,48)_ = 0.93; *p* = 0.48). Shown is the daily mean escape latency (seconds ± SEM), i.e., the time animals spent to reach the hidden platform throughout the 7-day-long training. V1 and V2 refer to the first two training sessions of day 1, carried out with a visible platform to rule out mouse sensorimotor deficits (not included in the analysis). Furthermore, no significant differences between groups were detected in averaged swimming speed (*p* = 0.947, Student’s *t* test) and thigmotaxis (*p* = 0.702 Student’s *t* test) during the whole training (data not plotted).
