# Supplementary material for: Terminal Differentiation of Adult Hippocampal Progenitor Cells Is a Step Functionally Dissociable from Proliferation and Is Controlled by Tis21, Id3 and NeuroD2
Source: Front Cell Neurosci. 2017 Jul 10;11:186. doi: 10.3389/fncel.2017.00186 (PMC5502263; doi:10.3389/fncel.2017.00186)
Supplement: Supplementary file 1 [file Table_1.docx]

| Experimental protocol | Neurogenic stimulus | Thymidine analogue injection | Sacrifice | n. mice per group | Cell staining | Measured variables and statistical test |
| --- | --- | --- | --- | --- | --- | --- |
| Timeline Fig. 1  (FLX - Proliferation Paradigm) | FLX (10mg/Kg)  18 days (P58🡪P75) |  | The day after last FLX injection (P76) | 4 Tis21^WT^+H20; 6 Tis21^WT^+FLX;  4 Tis21^KO^+H2O; 5 Tis21^KO^+FLX | Ki67/Nestin/DCX | Cell number in whole DG (Mean ± SEM). ANOVA and PLSD test. |
| Timeline Fig. 2B  (FLX - cell cycle length) | FLX (10mg/Kg)  21 days (P39🡪P59) | Idu (57.5mg/Kg) and CldU (42.5mg/Kg) the day after last FLX injection(P60) | The day after last FLX injection (P60) | 5 Tis21^WT^+H20; 5 Tis21^WT^+FLX;  5 Tis21^KO^+H2O; 5 Tis21^KO^+FLX  4 Tis21^WT^+H20; 3 Tis21^WT^+FLX;  4 Tis21^KO^+H2O; 3 Tis21^KO^+FLX  (are the same mice used for IdU/CldU count) | IdU/CldU  IdU/CldU/ND1 | Ts (Hours ± SEM). ANOVA and PLSD test.  Ts (Hours ± SEM). ANOVA and PLSD test. |
| Timeline Fig. 2E  (cell cycle length) |  | Idu (57.5mg/Kg) and CldU (42.5mg/Kg) (P14) | P14 | 5 Tis21^WT^ ; 5 Tis21^KO^ | IdU/CldU  IdU/CldU/ND1 | Ts (Hours ± SEM) Student’s t test |
| Data not shown  (FLX - Proliferation Paradigm) | FLX (10mg/Kg)  18 days (P60🡪P77) | Daily injections of BrdU (95mg/Kg) during the last 5 days of FLX treatment (P73🡪P77) | The day after last FLX injection (P78) | 3 Tis21^WT^+H20; 3 Tis21^WT^+FLX;  3 Tis21^KO^+H2O; 3 Tis21^KO^+FLX | BrdU/CR/NeuN | Cell number in whole DG (Mean ± SEM). ANOVA test and PLSD test.  Cell Ratio to total BrdU (Mean ± SEM); Kruskall-Wallis and Mann-Whitney U test |
| Timeline Fig.S1B  (FLX - Proliferation/Differentiation Paradigm) | FLX (10mg/Kg)  18 days (P60🡪P77) | Daily injections of BrdU (95mg/Kg) during the last 5 days of FLX treatment (P73🡪P77) | 10 days after end of FLX treatment  (P88) | 4 Tis21^WT^+H20; 4 Tis21^WT^+FLX;  4 Tis21^KO^+H2O; 4 Tis21^KO^+FLX | BrdU/CR/NeuN | Cell number in whole DG (Mean ± SEM); ANOVA and PLSD test.  Cell Ratio to total BrdU (Mean ± SEM); Kruskall-Wallis and Mann-Whitney U test |
| Timeline Fig. S2B  (7 days FLX - Proliferation Test) | FLX (10mg/Kg)  7 days (P60🡪P66) | Daily injections of BrdU (95mg/Kg) during the first 5 days of FLX treatment (P60🡪P64) | The day after last FLX injection (P67) | 5 Tis21^WT^+H20; 5 Tis21^WT^+FLX; | BrdU | Cell number in whole DG (Mean ± SEM)  ANOVA and PLSD test. |
| Timeline Fig. 3B  (FLX - Proliferation/Differentiation/Survival Paradigm) | FLX (10mg/Kg)  18 days (P60🡪P77) | Daily injections of BrdU (95mg/Kg) during the first 5 days of FLX treatment (P60🡪P64) | 10 days after end of FLX treatment  (P88) | 3 Tis21^WT^+H20; 3 Tis21^WT^+FLX;  3 Tis21^KO^+H2O; 3 Tis21^KO^+FLX | BrdU/CR/NeuN | Cell number in whole DG (Mean ± SEM); ANOVA and PLSD test. Cell Ratio to total BrdU (Mean ± SEM); Kruskall-Wallis and Mann-Whitney U test |
| Timeline Fig. 4B  (MWM - Differentiation/Survival Paradigm) | MWM  1 week after BrdU injections (P68🡪P74) | 2 daily injections of BrdU (95mg/Kg) at the beginning of protocol for two days (P60-P61) | 21 days after BrdU injections  (P81) | 5 Control Tis21^WT^; 5 Trained Tis21^WT^;  5 Control Tis21^KO^; 5 Trained Tis21^KO^  (two one-in eight series analyzed per mouse) | BrdU/CR/NeuN | Cell number in whole DG (Mean ± SEM); ANOVA and PLSD test.  Cell Ratio to total BrdU (Mean ± SEM); Kruskall-Wallis and Mann-Whitney U test. |
| Timeline Fig. 5A-6A-7A  (Retrovirus infection –Proliferation & Differentiation) | GFP-retrovirus injections (P60) |  | 5 days after injection (P65) | 3 Tis21^ko^ Injected with empty virus (control)  3 Tis21^ko^ Injected with expression virus | GFP/Ki67  GFP/CR/NeuN | Percentage ratio to total GFP positive cells (Mean ± SEM). Mann-Whitney U test |

**Table S1.** Summary of the experiments performed: protocols, mice number used and analyses accomplished.
