# Supplementary material for: Terminal Differentiation of Adult Hippocampal Progenitor Cells Is a Step Functionally Dissociable from Proliferation and Is Controlled by Tis21, Id3 and NeuroD2
Source: Front Cell Neurosci. 2017 Jul 10;11:186. doi: 10.3389/fncel.2017.00186 (PMC5502263; doi:10.3389/fncel.2017.00186)
Supplement: Supplementary file 2 [file Table_2.docx]

Table S2. Two-way ANOVA analyses of experiments with two-factors

| Experiment | Two-way ANOVA  factors | Two-way ANOVA  DF | Two-way ANOVA F-Value | Two-way ANOVA  P-Value | Fisher’s PLSD Post-hoc comparisons |
| --- | --- | --- | --- | --- | --- |
| Figure 1C  *Ki67* | Genotype  Treatment  Genotype × Treatment | 1,143  1,143  1,143 | 12.492  31.800  0.553 | 0.0006  <0.0001  0.4582 | Tis21KO-CTL vs Tis21KO-FLX 0.0003  Tis21KO-CTL vs Tis21WT-CTL 0.0051  Tis21KO-CTL vs Tis21WT-FLX 0.1241  Tis21KO-FLX vs Tis21WT-CTL <0.0001  Tis21KO-FLX vs Tis21WT-FLX 0.0363  Tis21WT-CTL vs Tis21WT-FLX <0.0001 |
| Figure 1D  *Type-1-2a* | Genotype  Treatment  Genotype × Treatment | 1,143  1,143  1,143 | 0.006  6.283  4.442 | 0.9397  0.0133  0.0368 | Tis21KO-CTL vs Tis21KO-FLX 0.7644  Tis21KO-CTL vs Tis21WT-CTL 0.1784  Tis21KO-CTL vs Tis21WT-FLX 0.0597  Tis21KO-FLX vs Tis21WT-CTL 0.0994  Tis21KO-FLX vs Tis21WT-FLX 0.1003  Tis21WT-CTL vs Tis21WT-FLX 0.0024 |
| Figure 1D  *Type-2b* | Genotype  Treatment  Genotype × Treatment | 1,143  1,143  1,143 | 0.670  13.272  0.182 | 0.4145  0.0004  0.6701 | Tis21KO-CTL vs Tis21KO-FLX 0.0168  Tis21KO-CTL vs Tis21WT-CTL 0.4086  Tis21KO-CTL vs Tis21WT-FLX 0.0397  Tis21KO-FLX vs Tis21WT-CTL 0.0028  Tis21KO-FLX vs Tis21WT-FLX 0.7672  Tis21WT-CTL vs Tis21WT-FLX 0.0073 |
| Figure 1D  *Type-3* | Genotype  Treatment  Genotype × Treatment | 1,143  1,143  1,143 | 23.539  15.653  0.049 | <0.0001  0.0001  0.8254 | Tis21KO-CTL vs Tis21KO-FLX 0.0020  Tis21KO-CTL vs Tis21WT-CTL 0.0025  Tis21KO-CTL vs Tis21WT-FLX 0.5116  Tis21KO-FLX vs Tis21WT-CTL <0.0001  Tis21KO-FLX vs Tis21WT-FLX 0.0002  Tis21WT-CTL vs Tis21WT-FLX 0.0136 |
| Figure 2C  *Ts*  *total progenitors* | Genotype  Treatment  Genotype × Treatment | 1,164  1,164  1,164 | 1.111  2.045  2.640 | 0.2933  0.1546  0.1061 | Tis21KO-CTL vs Tis21KO-FLX 0.0207  Tis21KO-CTL vs Tis21WT-CTL 0.7615  Tis21KO-CTL vs Tis21WT-FLX 0.8934  Tis21KO-FLX vs Tis21WT-CTL 0.0337  Tis21KO-FLX vs Tis21WT-FLX 0.0563  Tis21WT-CTL vs Tis21WT-FLX 0.8960 |
| Figure 2D  *Ts NeuroD1 progenitors* | Genotype  Treatment  Genotype × Treatment | 1,102  1,102  1,102 | 1.418  1.100  0.317 | 0.2365  0.2966  0.5744 | Tis21KO-CTL vs Tis21KO-FLX 0.7132  Tis21KO-CTL vs Tis21WT-CTL 0.2324  Tis21KO-CTL vs Tis21WT-FLX 0.9287  Tis21KO-FLX vs Tis21WT-CTL 0.0702  Tis21KO-FLX vs Tis21WT-FLX 0.6471  Tis21WT-CTL vs Tis21WT-FLX 0.2864 |
| Figure 3C  *Stage 5* | Genotype  Treatment  Genotype × Treatment | 1,80  1,80  1,80 | 12.679  18.446  0.241 | 0.0006  <0.0001  0.6250 | Tis21KO-CTL vs Tis21KO-FLX 0.0013  Tis21KO-CTL vs Tis21WT-CTL 0.0284  Tis21KO-CTL vs Tis21WT-FLX 0.5811  Tis21KO-FLX vs Tis21WT-CTL <0.0001  Tis21KO-FLX vs Tis21WT-FLX 0.0066  Tis21WT-CTL vs Tis21WT-FLX 0.0076 |
| Figure 3C  *Stage 6* | Genotype  Treatment  Genotype × Treatment | 1,79  1,79  1,79 | 39.645  72.355  11.140 | <0.0001  <0.0001  0.0013 | Tis21KO-CTL vs Tis21KO-FLX 0.0004  Tis21KO-CTL vs Tis21WT-CTL 0.0347  Tis21KO-CTL vs Tis21WT-FLX <0.0001  Tis21KO-FLX vs Tis21WT-CTL 0.1497  Tis21KO-FLX vs Tis21WT-FLX <0.0001  Tis21WT-CTL vs Tis21WT-FLX <0.0001 |
| Figure 4C  *Stage 5* | Genotype  Treatment  Genotype × Treatment | 1,270  1,270  1,270 | 38.530  27.176  7.532 | <0.0001  <0.0001  0.0065 | Tis21KO-CTL vs Tis21KO-MWM <0.0001  Tis21KO-CTL vs Tis21WT-CTL 0.0158  Tis21KO-CTL vs Tis21WT-MWM 0.5002  Tis21KO-MWM vs Tis21WT-CTL <0.0001  Tis21KO-MWM vs Tis21WT-MWM<0.0001  Tis21WT-CTL vs Tis21WT-MWM 0.0421 |
| Figure 4C  *Stage 6* | Genotype  Treatment  Genotype × Treatment | 1,270  1,270  1,270 | 7.251  15.354  0.022 | 0.0075  0.0001  0.8033 | Tis21KO-CTL vs Tis21KO-MWM 0.0113  Tis21KO-CTL vs Tis21WT-CTL 0.0475  Tis21KO-CTL vs Tis21WT-MWM <0.0001  Tis21KO-MWM vs Tis21WT-CTL 0.3659  Tis21KO-MWM vs Tis21WT-MWM 0.0705  Tis21WT-CTL vs Tis21WT-MWM 0.0020 |
| Figure S1C  *Stage 5* | Genotype  Treatment  Genotype × Treatment | 1,121  1,121  1,121 | 10.037  11.742  0.087 | 0.0019  0.0008  0.7681 | Tis21KO-CTL vs Tis21KO-FLX 0.0099  Tis21KO-CTL vs Tis21WT-CTL 0.0403  Tis21KO-CTL vs Tis21WT-FLX 0.8558  Tis21KO-FLX vs Tis21WT-CTL <0.0001  Tis21KO-FLX vs Tis21WT-FLX 0.0178  Tis21WT-CTL vs Tis21WT-FLX 0.0281 |
| Figure S1C  *Stage 6* | Genotype  Treatment  Genotype × Treatment | 1,121  1,121  1,121 | 23.044  15.236  0.016 | <0.0001  0.0002  0.9000 | Tis21KO-CTL vs Tis21KO-FLX 0.0053  Tis21KO-CTL vs Tis21WT-CTL 0.0005  Tis21KO-CTL vs Tis21WT-FLX <0.0001  Tis21KO-FLX vs Tis21WT-CTL 0.5255  Tis21KO-FLX vs Tis21WT-FLX 0.0015  Tis21WT-CTL vs Tis21WT-FLX 0.0084 |
| Figure S3  *Escape latency* | Genotype  Training  Genotype × Training | 1,48  6,48  6,48 | 1,865  10,260  0,934 | 0,209  <0.0001  0,479 | D1-Tis21WT vs D2-Tis21WT 0.0486  D1-Tis21WT vs D3-Tis21WT 0.0062  D1-Tis21WT vs D4-Tis21WT 0.0022  D1-Tis21WT vs D5-Tis21WT 0.0002  D1-Tis21WT vs D6-Tis21WT 0.0011  D1-Tis21WT vs D7-Tis21WT 0.0003  D1-Tis21KO vs D2-Tis21KO 0.6734 D1-Tis21KO vs D3-Tis21KO 0.1226  D1-Tis21KO vs D4-Tis21KO 0.0021 D1-Tis21KO vs D5-Tis21KO 0.0002 D1-Tis21KO vs D6-Tis21KO 0.00003  D1-Tis21KO vs D7-Tis21KO 0.00005 |
