# Supplementary figures and images for: Terminal Differentiation of Adult Hippocampal Progenitor Cells Is a Step Functionally Dissociable from Proliferation and Is Controlled by Tis21, Id3 and NeuroD2
Source: Front Cell Neurosci. 2017 Jul 10;11:186. doi: 10.3389/fncel.2017.00186 (PMC5502263; doi:10.3389/fncel.2017.00186)

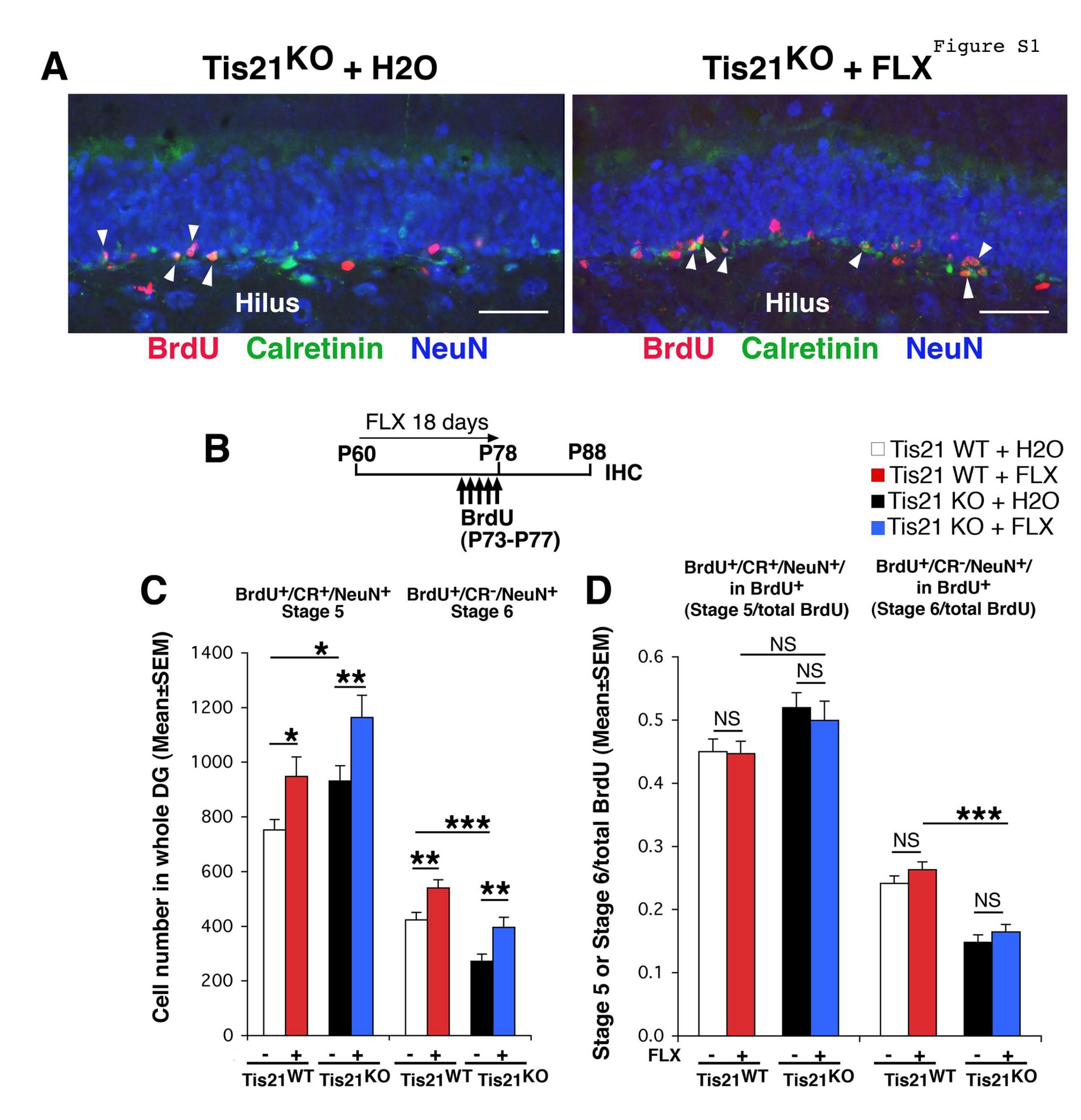

Supplement: FIGURE S1 — Fluoxetine, administered with a protocol highlighting proliferation and differentiation, rescues stage 5 but not stage 6 neurons defective for terminal differentiation in Tis21 knockout mice. (A) Representative confocal images (40×) showing stage 5 (BrdU+/Calretinin+/NeuN+; white arrowheads), and stage 6 neurons, (BrdU+/Calretinin−/NeuN+), treated as described in (B). Scale bar, 100 μm. (B) Two-month-old mice received five daily injection of BrdU at the end of the fluoxetine treatment, followed by 10 days off treatment to allow differentiation. (C) Quantification of the number of new 15-day-old cells shows increase in Tis21-null dentate gyrus of stage 5 immature neurons and decrease of terminally differentiated stage 6 neurons, relative to wild-type. Fluoxetine increases both stage 5 and stage 6 neurons in wild-type and Tis21 knockout dentate gyrus. Simple effects analysis: *p < 0.05, **p < 0.01, or ***p < 0.001; PLSD ANOVA test. Cell numbers in the dentate gyrus are mean ± SEM of the analysis of four animals per group. (D) The ratio of stage 5 neurons to the total number of BrdU+ cells was restored by fluoxetine in mutant cells to the values of wild-type cells; nevertheless, no rescue was observed for fluoxetine-treated stage 6 mutant neurons that remained lower than control. Simple effects analysis: NS, p > 0.05, ***p < 0.001; Mann-Whitney U test. [file Image_1.jpg]

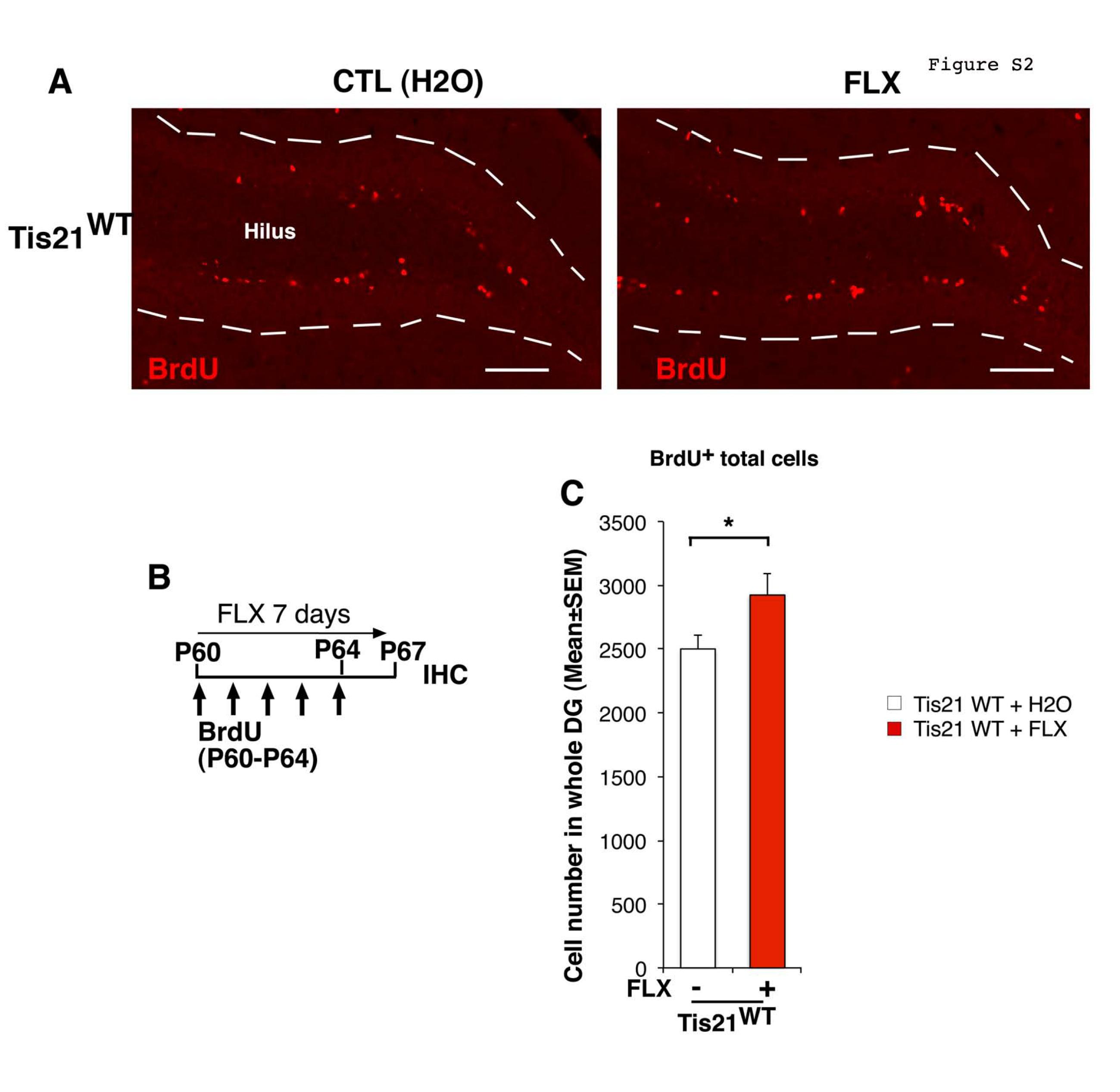

Supplement: FIGURE S2 — Fluoxetine treatment induces after 7 days a significant increase of the number of dentate gyrus progenitor cells in adult wild-type mice, as detected following multiple BrdU injections. (A) Representative confocal images (40× magnification) showing proliferating dentate gyrus progenitor cells, labeled by BrdU (red), in mice treated as described in (B). Dotted lines delimit the outer boundary of the granule cell layer. Scale bar, 100 μm. (B) Two-month-old mice received five daily injection of BrdU at the beginning of the fluoxetine treatment, which lasted 7 days. (C) Quantification of total proliferating adult progenitor cells in wild-type dentate gyrus, measured as BrdU+ cells. Cell numbers in the dentate gyrus are mean ±SEM of the analysis of five animals per group. *p < 0.05; PLSD ANOVA test. [file Image_2.jpg]

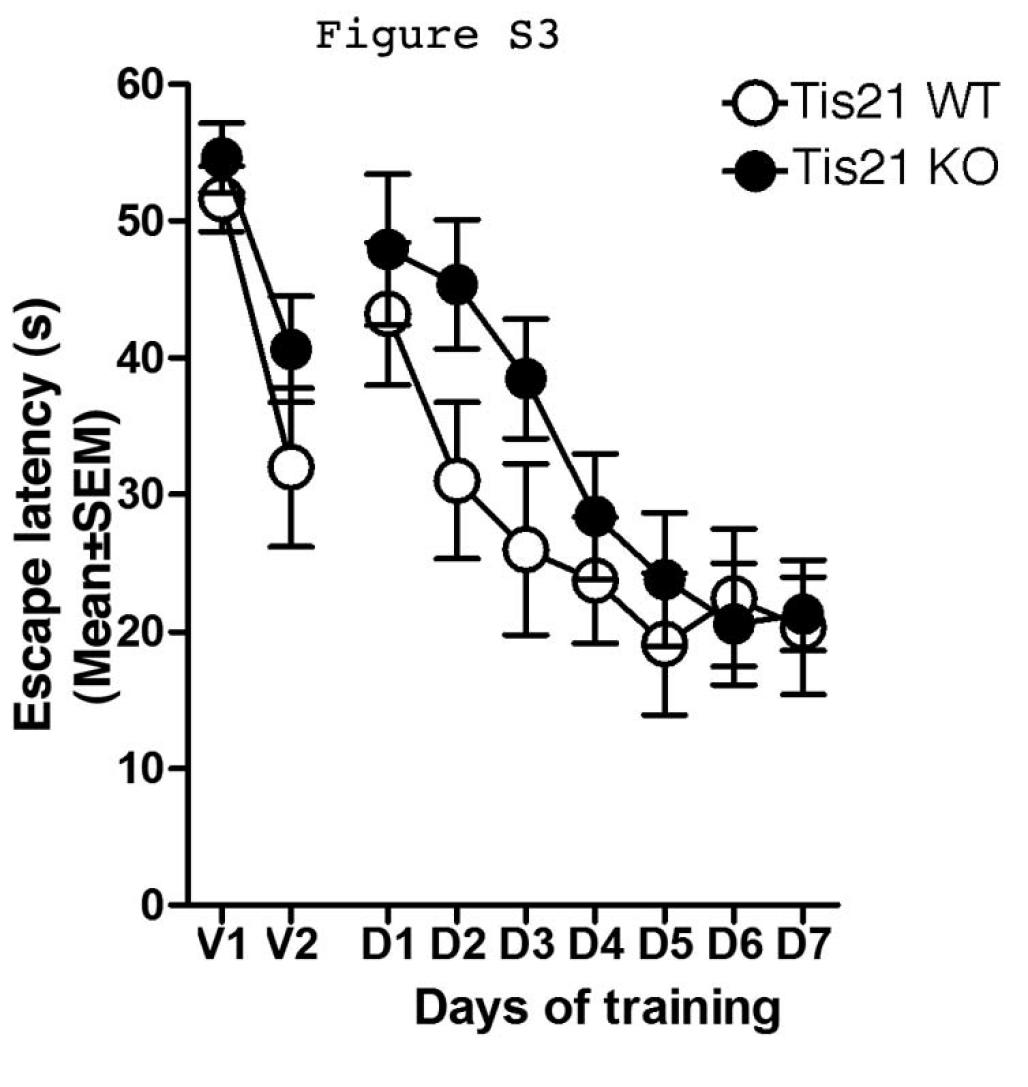

Supplement: FIGURE S3 — Morris water maze (MWM) escape latency in wild-type and mutant mice. The MWM was performed as in Farioli-Vecchioli et al. (2009) with minor modifications. In this task, mice learn across daily sessions to find a hidden escape platform using extra-maze visual cues. Tis21KO and Tis21WT mice (both groups, n = 5) performed equally in the task. Statistical analysis (repeated measures ANOVA) showed a significant effect of training (F(6,48) = 10.26; p < 0.0001), no significant effect of genotype (F(1,8) = 1.87; p = 0.21) and no significant genotype × training interaction (F(6,48) = 0.93; p = 0.48). Shown is the daily mean escape latency (seconds ± SEM), i.e., the time animals spent to reach the hidden platform throughout the 7-day-long training. V1 and V2 refer to the first two training sessions of day 1, carried out with a visible platform to rule out mouse sensorimotor deficits (not included in the analysis). Furthermore, no significant differences between groups were detected in averaged swimming speed (p = 0.947, Student’s t test) and thigmotaxis (p = 0.702 Student’s t test) during the whole training (data not plotted). [file Image_3.jpg]
